# Fabrication, Characterization and Evaluation of an Alginate–Lignin Composite for Rare-Earth Elements Recovery

**DOI:** 10.3390/ma15030944

**Published:** 2022-01-26

**Authors:** Dominika Fila, Zbigniew Hubicki, Dorota Kołodyńska

**Affiliations:** Department of Inorganic Chemistry, Faculty of Chemistry, Institute of Chemical Sciences, Maria Curie-Skłodowska University, Maria Curie-Skłodowska Sq. 2, 20-031 Lublin, Poland; zbigniew.hubicki@mail.umcs.pl

**Keywords:** alginate, lignin, composite, adsorption, reusability

## Abstract

The recent increase in interest in rare earth elements is due to their increasing use in many areas of life. However, along with their increasing popularity, the problem of their natural resources availability arises. In this study, an alginate–lignin composite (ALG-L) was fabricated and tested for adsorptive abilities of the rare earth elements (La(III), Ce(III), Pr(III), and Nd(III)) from aqueous solutions. The characterization of the newly synthetized calcium alginate–lignin composite was performed using ATR/FT-IR, SEM, EDX, OM, AFM, XRD, BET, sieve analysis and pH_pzc_ measurements. The adsorption mechanism of the ALG_5_L_1_ composite for REEs was analyzed through a series of kinetic, equilibrium and thermodynamic adsorption experiments. Under the optimum sorption conditions, i.e., sorbent mass 0.1 g, pH 5.0, temperature 333 K, the maximum adsorption capacities of the ALG_5_L_1_ composite for La(III), Ce(III), Pr(III), and Nd(III) reached 109.56, 97.97, 97.98, and 98.68 mg/g, respectively. The desorption studies indicate that the new calcium alginate–lignin composite is characterized by good recycling properties and can be also reused. To sum up the advantages of low cost, easy synthesis, high adsorption efficiencies and reusability indicate that the ALG_5_L_1_ composite has great application perspectives for REEs recovery.

## 1. Introduction

Nowadays, rare earth elements (REEs) are of great interest because of their enormous potential for many technological applications. They are widely used in electronics, medical equipment, fluorescent and LED lighting and digital cameras, among others [1,2,3]. For example, pure lanthanum is used to make lenses, microscopes, hard drives in computers, catalysts, super alloys, and also nickel-metal hydride batteries, which have replaced the more environmentally burdensome nickel–cadmium batteries [4]. Europium is responsible for the red colour displayed on the TV screen while neodymium and dysprosium are used in hybrid cars. Gadolinium is a component of energy-saving fluorescent lamps and cerium oxide is used in the production of optical instruments. Yttrium, erbium and terbium are used in microwave ovens, energy-saving light bulbs and as alloying additives for stainless steel. Due to such numerous applications, the demand for rare earth elements is increasing. The global rare earth market is dominated by China. Instead of developing the production of rare earth elements and their compounds to meet the ever-increasing demand for them, China is systematically restricting their exports. Yttrium, thulium and terbium have been placed under a total export ban, and in the case of neodymium, lanthanum, cerium and europium their exports are restricted. The policy of Chinese authorities is aimed at ensuring that technologies based on the use of the above-mentioned critical elements are developed and applied in China [1,5]. For this reason, the recovery of rare earth elements from worn-out magnets (Nd-Fe-B, SmCo_5_, Sm(Co,Fe,Cu,Zn)_6.8–8.5_, fluorescent lamps and nickel-hydride batteries) is becoming increasingly common in a number of countries, including the USA, Japan and Germany [6,7,8,9,10]. The issue of rare earth elements recovery from technological solutions coming from the processing of the above-mentioned waste materials is very important for modern technology and industry. So far, recovery of metals from secondary sources has been done by traditional pyrometallurgical or hydrometallurgical methods. However, due to the great chemical similarity of rare earth elements and the formation of isomorphic mixtures, the separation process is extremely challenging. Based on the analysis of PubMed search results by Kang et al. [11], it is expected that REEs will remain a major topic in the environmental science.

Most papers are focused on physicochemical methods for their recovery such as solvent extraction, precipitation, co-precipitation, electrochemical and membrane processes, ion exchange and adsorption. There are different sorbent materials used for the REEs removal from the aqueous solutions such as, *Sargassum* biomass [12], granular hydrogel composite [13], cysteine-functionalized chitosan magnetic particles [14], modified red clays [15], and modified silica gels [16]. As an example of effective biosorbents with significance in the rare earth elements or heavy metal ions removal are alginate salts or their composites [17,18,19]. For example, Khotimchenko et al. [17] investigated the ability of calcium alginate and sodium alginate to remove yttrium ions from the aqueous solutions. The maximum adsorption capacities of calcium alginate and sodium alginate for Y(III) ions were 99.01 and 181.81 mg/g, respectively, at pH 6.0.

Researchers’ attempts are oriented to the search for efficient adsorbents that can find application in the rare earth elements recovery. In this study, the alginate–lignin composite (ALG-L) was fabricated using sodium alginate as a matrix and lignin as a dispersed phase by external gelation with calcium chloride as a crosslinking agent. Then, the ALG-L composite was tested for adsorptive abilities of the rare earth elements from the aqueous solutions. The preparation and coating of lignin on the alginate surface was confirmed and characterized using ATR/FT-IR spectroscopy. Additionally, the surface properties of the calcium alginate–lignin composite was analyzed by SEM with EDX (Scanning Electron Microscopy with Energy Dispersive X-ray spectroscopy), OM (Optical Microscopy), AFM (Atomic Force Microscopy), XRD (X-ray Powder Diffraction), BET surface area, sieve analysis and pH_pzc_ measurements. The effects of some experimental conditions, i.e., solution pH, composite mass, contact time, temperature, initial La(III), Ce(III), Pr(III) and Nd(III) concentrations, and the presence of interfering ions was studied. The Langmuir, Freundlich, Temkin, and Dubinin–Raduschkevich equations were used to fit the equilibrium data. Five kinetic models, i.e., pseudo-first order (PFO), pseudo-second order (PSO), Weber–Morris intraparticle diffusion (IPD), Boyd, and Dumwald–Wagner (DW) were selected for the kinetic investigations. Adsorption thermodynamics and regeneration studies of the alginate–lignin composite were also evaluated and discussed. The analogous experimental sorption studies were also carried out for raw lignin and finally compared with the calcium alginate–lignin composite. 

## 2. Materials and Methods

The various types of materials and chemicals used in the present study are as follows; i.e., the sodium salt of alginic acid from brown algae was obtained from Carl Roth (Karlsruhe, Germany). Alkali lignin was purchased from Sigma Aldrich (Steinheim, Germany). The hexahydrate nitrate salts of rare earth elements (i.e., La(NO_3_)_3_·6H_2_O, Ce(NO_3_)_3_·6H_2_O, Pr(NO_3_)_3_·6H_2_O, Nd(NO_3_)_3_·6H_2_O) were obtained commercially from Sigma Aldrich (Germany). The other chemicals, anhydrous calcium chloride, sodium hydroxide, nitric acid (65%), and hydrochloric acid (35–38%) were provided by Chempur (Piekary Śląskie, Poland) and silver nitrate was purchased from Sigma Aldrich (Germany). 1000 mg/dm^3^ of stock solution in a single component systems of La(III), Ce(III), Pr(III), and Nd(III) ions was prepared by adding a suitable amount of hexahydrate nitrate salts of La(III), Ce(III), Pr(III), and Nd(III) and distilled water into 1000 cm^3^ of volumetric flasks. After that the prepared solution was applied for preparation of various concentrations of La(III), Ce(III), Pr(III), and Nd(III) ions solution using the standard dilution method (C_1_V_1_ = C_2_V_2_). The solution pH was adjusted employing 0.1 mol/dm^3^ of HNO_3_ and NaOH aqueous solutions throughout all the experiments depending on requirements.

### 2.1. Optimization of the Alginate–Lignin Composite Fabrication

For optimization of the alginate–lignin composite fabrication different amounts of lignin were added to the alginate homogeneous solution. Firstly, 1% of alginate (named as ALG) solution was prepared by dissolving the sodium alginate powder in the deionized water under stirring at 500 rpm for 1 h at room temperature using an electromagnetic stirrer (ES 21, Wigo, Pruszków, Poland). Next, different amounts of lignin (named as L) were added to the 1% sodium alginate solution according to the chosen ratios between ALG and L and the mixture was mixed intensively for 1 h. Next, the obtained mixture was dropped into the 1% CaCl_2_ solution using the BT100S-1 peristaltic pump with the pump head YZ15 at a flow rate 1 cm^3^/min (Lead Fluid Technology Co., Ltd.,Baoding, Hebei, China). After 24 h the composite beads were thoroughly rinsed with deionized water to remove the CaCl_2_ excess. The prepared composite beads were named as: L (0:1, ALG:L), ALG_5_L_1_ (5:1, ALG:L), ALG_2_L_1_ (2:1, ALG:L), ALG_1_L_1_ (1:1, ALG:L), ALG_1_L_2_ (1:2, ALG:L), and ALG_1_L_5_ (1:5, ALG:L). The preliminary studies of La(III), Ce(III), Pr(III), and Nd(III) ions sorption on the fabricated alginate–lignin composite were carried out and the obtained results are presented in Figure S1 (see Supplementary Materials). 

The highest values of sorption efficiency (%S) were obtained at the ALG:L ratios equal 5:1 and 2:1. The further lignin mass increase resulted in a decrease in sorption effectiveness, possibly due to blocking of active sorption sites present in the composites structure. Hence, for further studies the alginate–lignin composite loaded with 20% of lignin particles named as ALG_5_L_1_ was chosen. 

### 2.2. Characterization of the Alginate–Lignin Composite

The prepared ALG-L composite was characterized using the ATR/FT-IR, SEM, EDX, OM, AFM, XRD, and BET techniques. The presence of probable functional groups in the alginate as well as the coating of lignin were identified by ATR/FT-IR (Cary 630, Agilent, Santa Clara, CA, USA). Then SEM (Quanta 3D FEG, FEI, Hillsboro, OR, USA) equipped with the energy-dispersive X-ray spectroscopy (EDX, FEI) as well as OM (AZ100M, Nikon, Tokyo, Japan) analyses were applied to scrutinize the morphologies, size, and shape of the fabricated ALG-L composite. Additionally, the surface mapping of the alginate–lignin composite was explored by Atomic Force Microscopy (AFM) (Veeco Nanoscope V, New York, NY, USA). The roughness average (R_a_) and the root mean square (R_q_) roughness were determined. The X-ray powder diffraction (XRD) patterns were recorded using a Bruker AXS D8 Advance diffractometer (equipped with the silicon strip LynxEye detector and Johansson monochromator) using Cu Kα radiation (λCu K_α1_ = 15,406 Å) with a step size of 0.01° in a 2θ range of 4–60° and the scanning rate of 0.6° min^−^^1^. Moreover, the surface properties of the ALG-L composite were estimated by Autosorb iQ Analyzer (Quantachrome, Graz, Austria) at 77 K using nitrogen gas. The samples were degassed at 353 K for over 24 h. The particle size distribution was performed by the sieve analysis using the Retsch laboratory sieves (Katowice, Poland) with the mesh sizes ranging from 900 to 1200 µm. Additionally, the point of zero charge of the fabricated composite was determined using the well-known drift method described in the literature. After the sorption process of La(III) ions possible interactions between the ALG-L composite and La(III) ions were analyzed and confirmed by the ATR/FT-IR, SEM, and EDX methods. 

### 2.3. Instrumentation and Methodology for Adsorption/Desorption Procedures

The batch adsorption experiments were made by shaking the Erlenmeyer flasks at a constant speed (180 rpm) and amplitude (8) in the atmospheric pressure and at ambient temperature (293 K) using a laboratory shaker (Elpin+ 458A, Poland); 20 cm^3^ of metal ions solution (25–500 mg/dm^3^) was added into the equal quantities of the ALG_5_L_1_ composite (0.05 g). The pH of the metal ions solution was adjusted to 5 unless otherwise stated by the pH-meter (PHM82 Radiometer, city Copenhagen, Denmark). The ALG_5_L_1_ beads were separated from the final solutions at the end of each adsorption or desorption experiment. Next, the final metal ions concentration was analyzed by the inductively coupled plasma optical emission spectrometry (720-ES Varian Inc., Palo Alto, CA, USA). La(III), Ce(III), Pr(III), and Nd(III) ions concentrations were estimated at the wavelengths of 333.749, 446.021, 410.072 and 401.224 nm, respectively. All the experiments were performed in triplicate and the averaged values were the final ones. Finally, the basic adsorption or desorption parameters (Equations (1)–(5)) were determined as follows:

(1) $$q_{t}=\left(C_{0}-C_{t}\right)\times \frac{V}{m}$$

(2) $$q_{e}=\left(C_{0}-C_{e}\right)\times \frac{V}{m}$$

(3) $$\%S=\frac{C_{0}-C_{t}}{C_{0}}\times 100\%$$

(4) $$\%D=\frac{C_{des}}{C_{0}-C_{e}}\times 100\%$$

(5) $$K_{d}=\frac{\left(C_{0}-C_{t}\right)}{C_{t}}\times \frac{V}{m}$$

where *q_t_* and *q_e_* are the adsorption capacities at time *t* [mg/g] and equilibrium [mg/g], respectively, *%S* and *%D* are the adsorption and desorption efficiencies, respectively, *K_d_* is the distribution coefficient [cm^3^/g], *C*_0_, *C_t_*, *C_e_*, and *C_des_* are the initial, after time *t,* at the equilibrium and after the desorption concentrations [mg/dm^3^], respectively, *V* is the solution volume [cm^3^], and *m* is the sorbent dose [g].

The sorption performance of the ALG_5_L_1_ composite was investigated by varying the process parameters as follows: the initial solution pH (1–7), dosage (0.05–0.5 g), temperature (293–333 K), initial La(III), Ce(III), Pr(III), and Nd(III) ions concentration (25–500 mg/dm^3^), and interaction time (0–1440 min). To compare the sorption properties, analogous tests were carried out for raw lignin.

At first, different pH values (from 1 to 7) were used to study the effect of initial solution pH on the sorption performance in the batch mode for which 20 cm^3^ of metal ions solution (100 mg/dm^3^) was added into 0.05 g of ALG_5_L_1_ composite and then shaking for 480 min. The solution pH was measured before and after the sorption process.

Next, the effect of composite dosages on the sorption efficiency was optimized at different initial dosages from 0.05 to 0.5 g in 20 cm^3^ of metal ions solution (100 mg/dm^3^) and shaking time 480 min. The pH value of the solution was chosen based on the above studies. 

Moreover, the effect of interfering ions on the La(III), Ce(III), Pr(III), and Nd(III) ions sorption process was detected by adding suitable amounts of various selected competing species like (NaNO_3_, NaCl, Na_2_SO_4_, Ni(NO_3_)_2_) to the test solutions (100 mg/dm^3^ of chosen REE ions and 100 mg/dm^3^ of selected competing species) using the optimum ALG_5_L_1_ composite mass. The prepared samples were shaken for 480 min and then analyzed for the presence of REE ions. 

#### 2.3.1. The Thermodynamic, Kinetic, and Isotherm Studies

The thermodynamic, kinetic and equilibrium studies of the La(III), Ce(III), Pr(III) and Nd(III) ions sorption process were carried out to determine the mechanism and possible interactions of metal ions with the newly formed composite. 

Thermodynamic studies were performed using 0.05 g of ALG_5_L_1_ composite, 20 cm^3^ of metal ions solution (100 mg/dm^3^), shaking time 480 min, and different temperatures, i.e., 293, 313 and 333 K. The thermodynamic parameters-entropy change *ΔS°* (kJ/mol·K), free energy change *ΔG°* (kJ/mol), and enthalpy change *ΔH°* (kJ/mol) were calculated employing the equations presented below:

(6) ΔG°=ΔH°−TΔS°

(7) $$\Delta G{}^{\circ}=-RT\ln K_{c}$$

(8) $$\frac{d\ln K_{c}}{d(1/T)}=-\frac{\Delta H{}^{\circ}}{R}$$

where: *K_c_* is the equilibrium partition coefficient [dm^3^/g] calculated as *q_e_*/*C_e_*.

Kinetic studies were carried out according to the following procedure: 0.05 g of ALG_5_L_1_ composite was added to each 100 cm^3^ flask and then 20 cm^3^ of metal ions solution (25–200 mg/dm^3^) was poured into the flask. These mixtures were placed in a laboratory shaker and were shaken for a specified period of time. The final La(III), Ce(III), Pr(III), and Nd(III) ions concentrations were measured at the end of 1, 3, 5, 7, 10, 30, 60, 120, 180, 240, 360, 480, 960 and 1440 min period. The sorption kinetic parameters were calculated using five kinetic models, i.e., the pseudo-first order (Lagergren), the pseudo-second order (Ho and McKay), the intraparticle diffusion (Weber and Morris), the Boyd and the Dumwald-Wagner models. The kinetic models equations are given in Table 1.

Finally, the sorption isotherm experiments were conducted according to the following procedure: 20 cm^3^ of suitable metal ions solution was inserted into a fixed amount of 0.05 g ALG_5_L_1_ composite. The La(III), Ce(III), Pr(III), and Nd(III) ions solution concentration was altered from 25 to 500 mg/dm^3^. The isotherm studies were carried out at three temperatures. i.e., 293, 313, and 333 K and shaking time 24 h. The final La(III), Ce(III), Pr(III), and Nd(III) ions concentration was measured using the ICP-OES spectrometer. For the investigation of the sorption equilibrium parameters, four isotherm models, i.e., Langmuir, Freundlich, Temkin, and Dubinin–Radushkevich isotherms were employed. These models allow to explain the metal ions interactions with the ALG_5_L_1_ composite. The isotherm models equations are given in Table 2.

In addition, the Chi-square (*Χ*^2^) error function was used for fitting the isotherm models:

(9) $$\chi^{2}=\sum \frac{{\left(q_{e,\exp}-q_{e,cal}\right)}^{2}}{q_{e,cal}}$$

where: *q_e,exp_* is the amount of rare earth ions adsorbed at equilibrium determined experimentally and *q_e,cal_* is the adsorption capacity determined from the isotherm model. The isotherm models give the best fitting when the *Χ*^2^ values are low (below 1) and the *R*^2^ values are close to 1. 

#### 2.3.2. Desorption and Reusability Studies

For the desorption experiments, the previously adsorbed La(III), Ce(III), Pr(III) and Nd(III) ions on the ALG_5_L_1_ composite (0.05 g) were transferred to a flask containing 20 cm^3^ of a desorbing agent such as 0.1 M HCl, 0.1 M HNO_3_, 0.1 M NaCl or distilled water. The mixture was shaken at 180 rpm using a laboratory shaker at room temperature for 480 min. After that, the best desorbing agent was chosen and for those experiments to find out the optimal desorption conditions were carried out. The effects of the desorbing agent concentration (0.1–2.0 M) and desorption time (1–480 min) on the desorption efficiency were investigated.

To explore the La(III), Ce(III), Pr(III), and Nd(III) ions removal and the potential of ALG_5_L_1_ composite reusability for the long-term applications, sorption and desorption cycles were examined using 0.1 M HNO_3_. In the desorption experiments, the adsorbent with the adsorbed metals that were eluted by the desorbing agent were thoroughly washed several times by distilled water to remove any desorbing agent traces and then mixed again with the solution containing suitable metal ions for the next adsorption cycle. This procedure was applied to six consecutive adsorption and desorption cycles. 

## 3. Results and Discussion

### 3.1. Characterization of the Alginate–Lignin Composite

**Figure 1:**
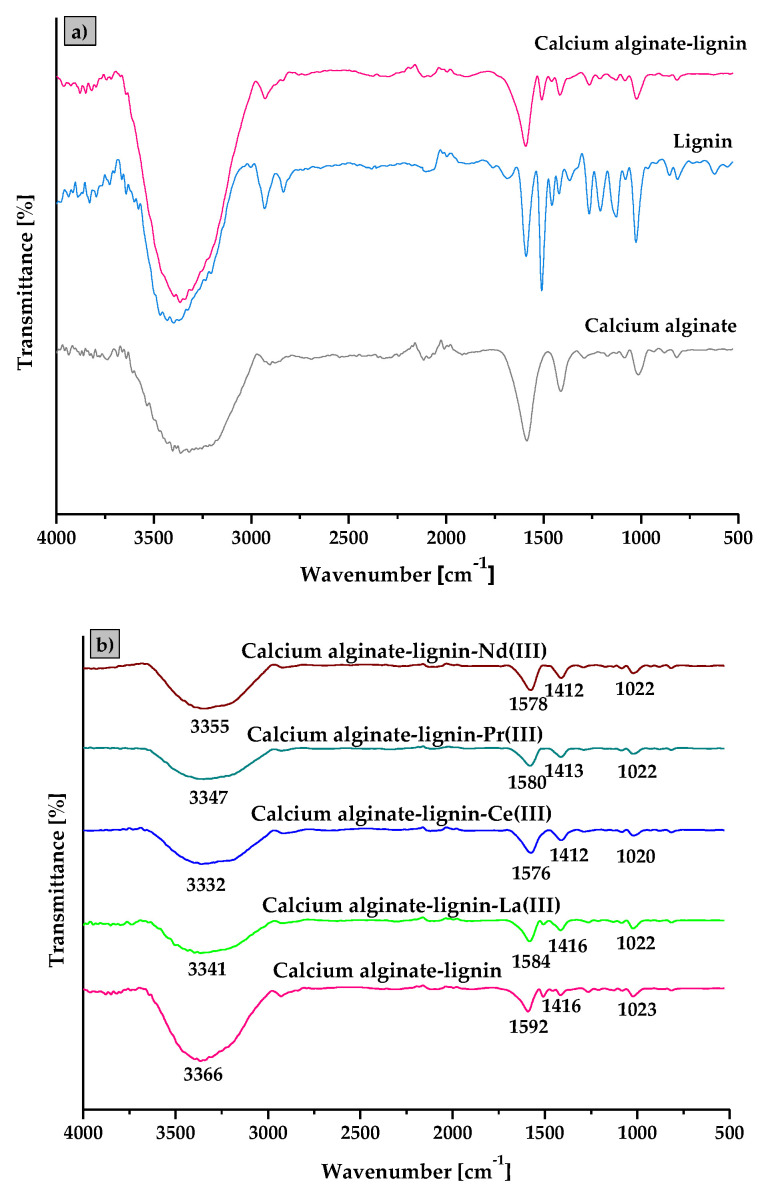
ATR/FT-IR of (**a**) calcium alginate, lignin, calcium alginate lignin composite and (**b**) before and after the La(III), Ce(III), Pr(III), and Nd(III) ions sorption on the calcium alginate–lignin composite.

The infrared spectra (ATR/FT-IR) analysis provides details about the sorbent structure especially the functional groups presence on the sorbent surface and allows to predict possible attachment of metal ions. The comparison of the Fourier transform infrared spectra of the calcium alginate–lignin composite as well as the raw calcium alginate and lignin is presented in Figure 1a. Additionally, the spectra after the La(III) ions sorption process on the calcium alginate–lignin composite are included in Figure 1b. 

The broad band located in the range of 3700–3000 cm^−1^ corresponding to the -OH stretching vibrations of hydroxyl functional groups and also the structural water content was detected in the calcium alginate, lignin as well as the new synthetized calcium alginate–lignin composite samples. In the 2900–3000 cm^−^^1^ range, a -CH stretching band appears for all sorbents. In the calcium alginate spectrum the strong peaks present at 1586 and 1412 cm^−^^1^ correspond to the C-O asymmetric and C-O symmetric stretching vibrations in the COO^−^ groups, respectively. Additionally, three characteristic bands of alginate located at 1172, 1084 and 1014 cm^−^^1^ were identified as C–O, C–O–C and C–C stretching vibrations [20,21]. In the lignin sample, there are many bands in the range of 1000–1700 cm^−^^1^ assigned to the C=O stretching vibrations (1590 cm^−^^1^), C=C stretching of aromatic ring (1510 cm^−^^1^), C–H deformation in –CH_2_– and –CH_3_ groups as well as C-H aromatic ring vibrations (1350–1450 cm^−^^1^), C–O stretching ring (1268 cm^−^^1^). Some characteristic bands assigned to the C–C, C–O and C=O stretching, aromatic C-H in-plane deformation as well as aromatic C-H in-plane deformation were detected at 1210, 1120 and 1020 cm^−1^ [22,23]. Bands characteristic of both calcium alginate and lignin appeared in the calcium alginate–lignin composite spectrum which indicates that lignin is effectively incorporated into the alginate structure. The spectra of ALG_5_L_1_ composite before and after the exposure to La(III), Ce(III), Pr(III), and Nd(III) ions prove a reaction between the carboxyl functional groups and the REEs (Figure 1b). After the reaction with La(III) ions, the peaks related to the carboxyl groups shifted from 1592 to 1584 cm^−1^ and were enhanced at 1416 and 1086 cm^−1^. Moreover, after the Ce(III), Pr(III), and Nd(III) ions sorption the peaks of COO^−^ groups were shifted from 1592 to 1576 cm^−^^1^ for Ce(III) ions, from 1592 to 1580 cm^−^^1^ for Pr(III) ions, and from 1592 to 1578 cm^−^^1^ for Nd(III) ions. These observations indicate that the sorption of REEs using ALG_5_L_1_ is due to the reaction with carboxyl groups.

**Figure 2:**
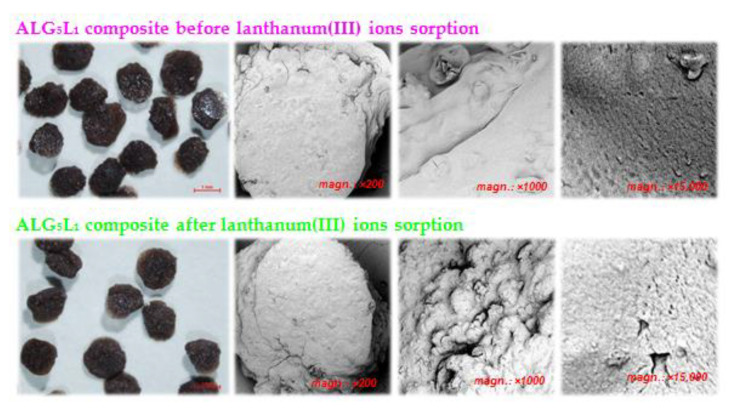
OM and SEM images of ALG_5_L_1_ composite before and after La(III) ions sorption at a magnification of 200, 1000, and 15,000×.

The ALG_5_L_1_ composite beads morphology and topography were analyzed using optical microscopy (OM) and scanning electron microscopy (SEM) both before and after the lanthanum(III) ions sorption process. The obtained images are shown in Figure 2.

The SEM analysis reveals the ALG_5_L_1_ composite porosity in the images with different magnifications (Figure 2). After the La(III) ions sorption, the surface became more wrinkled, rough and porous. Moreover, after the sorption process, the composite beads become more matte (opaque) as shown by the optical microscopy. 

In addition, the SEM analysis was made with EDX to get to know the chemical composition of the samples, i.e., elemental identification and their quantitative composition information (Table 1). The EDX analysis was performed over the areas of around 149 × 149 µm^2^ at a beam accelerating voltage equal 20 kV. Table 3 reports the chemical composition determined by SEM-EDX of the ALG_5_L_1_ composite before and after the La(III) ions sorption.

It should be noted that carbon, oxygen, and calcium are major constituents in the composite, additionally, there are small amounts of sulphur in the composite from lignin. The trace amounts of chlorine before the sorption process were due to their incomplete removal after the synthesis process. After the sorption process lanthanum appearance confirmed La(III) ions binding on the ALG_5_L_1_ surface. It should be mentioned that after the La(III) ions sorption the amount of calcium decreases from 7.96% to 1.00%, which confirms the ion exchange process. 

**Figure 3:**
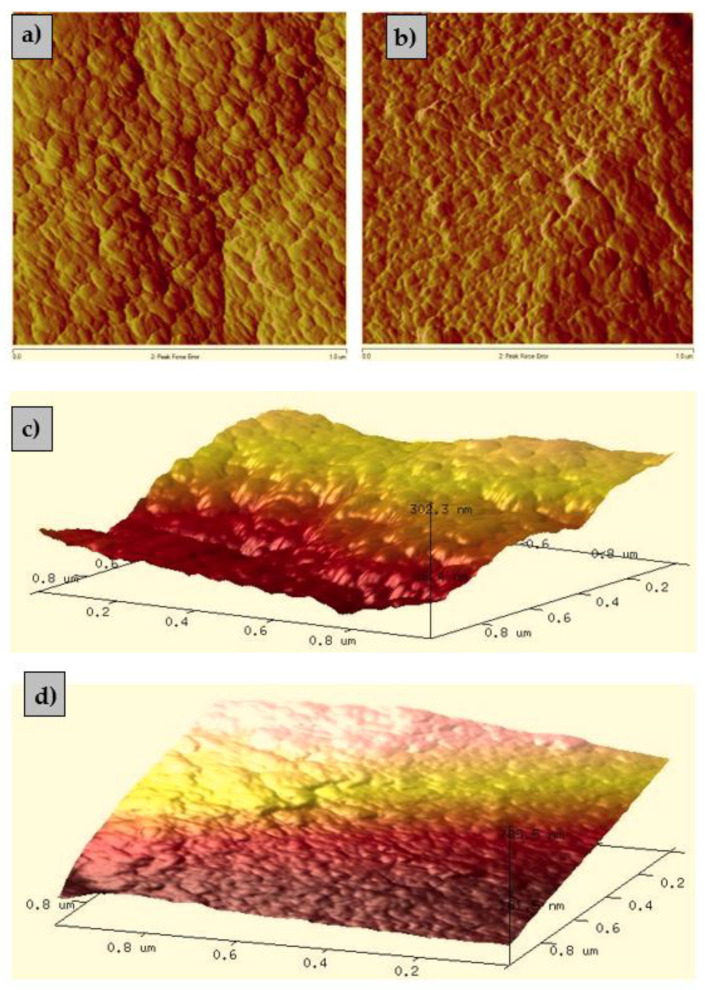
(**a**,**b**) 2D and (**c**,**d**) 3D AFM images before and after the La(III) ions sorption on the ALG_5_L_1_ composite.

Atomic force microscopy (AFM) was used to collect information about the topographical features of the composite such as morphology and surface roughness. The images shown in Figure 3 depict the 2D and 3D topographical comparison of the ALG_5_L_1_ composite before and after the La(III) ions sorption.

The AFM images of the calcium alginate–lignin composite show a relatively smooth surface. As follows from the 3D images the ALG_5_L_1_ composite (Figure 3c,d) is characterized by a more uneven surface with major trough features indicating that the surface roughness is increased compared to this composite after the La(III) ions sorption. The sorbent surface roughness is larger before the sorption process but after the sorption the porous structure is filled resulting in a smoother structure. The determined *R_a_* and *R_q_* roughness values were 24.8 and 31.3 nm before the La(III) ions sorption and 22.1 and 29.5 nm after the La(III) ions sorption.

The X-ray powder diffraction patterns were analyzed to study the morphology of calcium alginate, lignin, and newly synthetized calcium alginate–lignin composite in more detail. The typical XRD patterns of the samples are shown in Figure S2.

Two major peaks at 13.16° and 22.38° for alginate and three peaks at 18.32°, 31.78° and 33.86° for lignin were observed as confirmed by the previous studies [24,25,26]. The characteristic peaks for the calcium alginate and lignin were observed in the new composite at 14.88°, 21.29° and 31.96°. All samples were characterized by the broadening in the diffraction spectrum indicating their amorphous nature.

Additionally, the sieve analysis was carried out to measure the particle size distribution of ALG_5_L_1_ composite. For this purpose, laboratory sieves with the mesh sizes ranging from 900 to 1200 µm were used. The obtained results of the sieve analysis showed that the ALG_5_L_1_ composite is characterized by mono-dispersity, the prepared beads are uniform in size. Nearly 99% of the composite beads are 1100 µm in size (only 1% of particle size are 1200 µm). This is also confirmed by the images taken using optical microscopy (Figure 2).

The surface properties of the calcium alginate–lignin composite, i.e., specific surface area, pores diameter, total pores volume as well as micropores volume were analyzed using the surface area analyzer (Quantachrome, Graz, Austria). The specific surface area of the ALG_5_L_1_ composite beads was measured using the Brunauer–Emmet–Teller (BET) method. The total pore volume of the biosorbents was estimated from the amount adsorbed at *p*/*p*_0_ = 0.99. The textural parameters of the ALG_5_L_1_ composite as well as the raw calcium alginate and raw lignin are presented in Table S1. The new synthetized calcium alginate–lignin composite possesses the largest specific surface area 8.018 m^2^/g compared with the raw calcium alginate (4.707 m^2^/g) and raw lignin (1.918 m^2^/g). After the calcium alginate modification with lignin, the specific surface area and the pore size distribution of the new ALG_5_L_1_ composite increased. The calculated pore diameter values of biosorbents in the range of 2.975–6.107 nm indicate the presence of mesopores in their structure.

### 3.2. Evaluation of the Influence of Different Sorption Conditions on the Sorption Performance

The pH value determines the degree of dissociation of the molecules present in the solution and the functional groups on the sorbents surface. Metal ions in the aqueous solutions occurring in different forms can be adsorb or precipitate on the adsorbent surface. Thus, the metal speciation is one of the important factors affecting the adsorption process. As follows from the speciation graphs provided by Basualto et al. [27], Bouyer et al. [28], and Park and Tavlaride [29], the concentrations of speciation forms metals such as M(OH)^2+^, M(OH)_2_^+^, M(OH)_3_ or M(OH)_4_^−^ at pH < 6 are too low to affect the sorption process of the dominant form M^3+^ (M = La^3+^, Ce^3+^, Pr^3+^ or Nd^3+^).

**Figure 4:**
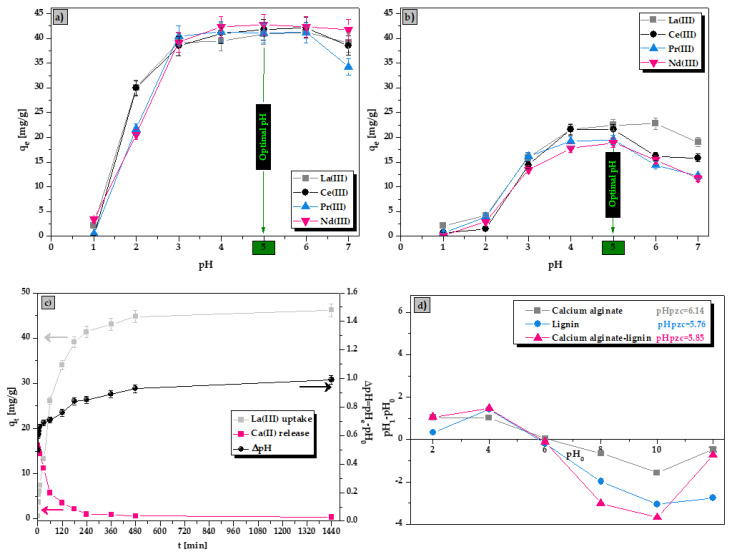
Influence of solution pH on the adsorption capacities of (**a**) ALG_5_L_1_ composite and (**b**) lignin, (**c**) La(III) uptake, Ca(II) release and pH changes during contact time, and (**d**) comparison of pH_pzc_ values of biosorbents (conditions: *pH* = 1–7, *C*_0_ = 100 mg/dm^3^, *m* = 0.05 g, *V* = 20 cm^3^, *t* = 480 min, *T* = 293 K).

To assess the effect of solution pH on the La(III), Ce(III), Pr(III), and Nd(III) ions sorption efficiency on the ALG_5_L_1_ composite and lignin the experiments in the pH range of 1–7 were conducted, keeping the initial metal ion concentration at 100 mg/dm^3^. The obtained results are presented in Figure 4a,b.

It was noticed that the change of solution pH influences on metal ions sorption. The La(III), Ce(III), Pr(III), and Nd(III) ions sorption does not occur at pH < 1, increases constantly in the rane of pH 2–5, and maintains a high level until pH = 6. In this pH range, the –COOH groups dissociated forming negatively charged –COO^−^ groups and were the main groups interacting with metal ions. At higher pH values (pH > 6), a decrease in the *q_e_* value was observed which may be due to the formation of metal hydroxides species. The surface of calcium alginate–lignin (ALG_5_L_1_) composite has the point of zero charge (pH_pzc_) at about 5.85 (Figure 4d). The optimum pH chosen for further sorption studies for the ALG_5_L_1_ composite was pH equal to 5, at which the *q_e_* values for La(III), Ce(III), Pr(III) and Nd(III) ions were 40.87 mg/g, 41.84 mg/g, 41.08 mg/g and 42.78 mg/g, respectively. On the other hand, the sorption efficiencies of La(III), Ce(III), Pr(III), and Nd(III) ions at pH 5 on the ALG_5_L_1_ composite were 99.29%, 99.28%, 99.40% and 97.72%, respectively. Comparing the above results to those of raw lignin, the optimum selected pH value was also pH 5 for which the sorption efficiency of La(III), Ce(III), Pr(III) and Nd(III) ions was 60.34%, 46.56%, 47.64% and 38.91%, respectively. 

In order to identify whether one mechanism of the sorption process is ion exchange, a parallel study of the kinetics of calcium release from the composite beads was also carried out (Figure 4c). The results indicate that there is an increase in pH (1 unit increase) during the sorption of La(III) ions on the ALG_5_L_1_ composite, resulting from the ion exchange of calcium present in the composite structure for lanthanum present in the solution. Additionally, as follows from Figure 4c, during the sorption of La(III) ions, Ca(II) ions are released from the composite structure, i.e., the concentration of La(III) ions decreases while that of Ca(II) ions increases in the aqueous solution. This confirms that one of the main sorption mechanisms of La(III) ions is ion exchange and the ALG_5_L_1_ composite behaves as a typical cation exchanger as shown below (where R is the composite structure):

(10) $$3{\left(-R-COO\right)}_{2}Ca+2La^{3+}\to 2{\left(-R-COO\right)}_{3}La+3Ca^{2+}$$

(11) $$3{\left(-R-O\right)}_{2}Ca+2La^{3+}\to 2{\left(-R-O\right)}_{3}La+3Ca^{2+}$$

The composite mass plays an important role in the sorption process because it determines the accessibility of active sites on the sorbent and thus the process efficiency. In this study, different amounts of the ALG_5_L_1_ composite (0.05–0.5 g) were employed to find out the optimal mass. Figure S3 illustrates the effect of sorbent mass on the La(III), Ce(III), Pr(III), and Nd(III) ions sorption efficiency as well as the sorption capacity of ALG_5_L_1_ composite. 

As the mass of the composite increases from 0.05 to 0.5 g, the number of unoccupied active sites on the sorbent increases, resulting in a decrease in the equilibrium capacity (*q_e_*) values. Moreover, the sorption efficiency was almost stable over the entire range of the tested mass and was close to 100% regardless of the sorbed metal ions. The optimum composite mass selected for further sorption studies was 0.05 g, at which the *q_e_* values for La(III), Ce(III), Pr(III) and Nd(III) ions were 41.23, 41.84, 41.08 and 41.98 mg/g, respectively. Analogous results were obtained for raw lignin for which, using a mass of 0.05 g, the following sorption capacities were obtained: 22.49 mg/g for La(III), 21.57 mg/g for Ce(III), 19.53 mg/g for Pr(III), and 18.90 mg/g for Nd(III). 

**Figure 5:**
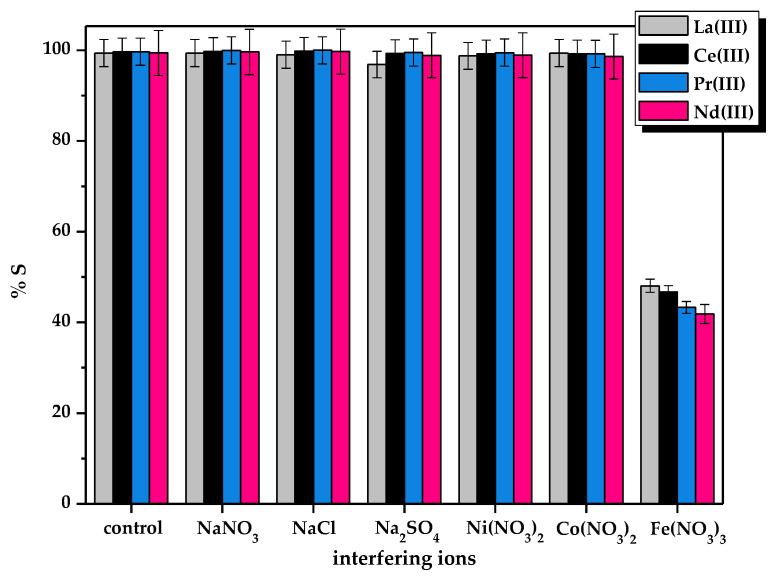
Effects of interfering ions on the La(III), Ce(III), Pr(III), and Nd(III) ions sorption using the ALG_5_L_1_ composite (conditions: *C*_0_ = 100 mg/dm^3^, *m* = 0.05 g, *V* = 20 cm^3^, *t* = 480 min, *T* = 293 K).

The prospective effect of interfering ions on the La(III), Ce(III), Pr(III), and Nd(III) ions sorption process onto the ALG_5_L_1_ composite was investigated. The adsorption performance values in the presence and absence of any interfering species such as NaNO_3_, NaCl, Na_2_SO_4_, Ni(NO_3_)_2_, Ni(NO_3_)_2_, as well as Fe(NO_3_)_3_ are presented in Figure 5.

The study revealed that the removal efficiency of La(III), Ce(III), Pr(III), and Nd(III) ions was altered mainly by the presence of Fe(III) ions. The effects of other species was not significant. There is a notable depreciation in the adsorption efficiency towards La(III), Ce(III), Pr(III), and Nd(III) ions due to the existence of interfering species, i.e., Fe(III) ions, more than 50% decrease in the adsorption efficiency of metal ions on the ALG_5_L_1_ composite was observed. The ALG_5_L_1_ composite showed higher affinity for metal ions in the third oxidation state rather than in the second and first oxidation ones.

### 3.3. The Thermodynamic, Kinetic, and Isotherm Studies 

Table S2 (see Supplementary Materials) presents the obtained thermodynamic parameters for the La(III), Ce(III), Pr(III), and Nd(III) ions sorption onto the ALG_5_L_1_ composite. The obtained negative values of *ΔG°* indicated the thermodynamically feasible spontaneous character of the metal ions sorption onto the ALG_5_L_1_ composite. Moreover, the *ΔG°* values increased with an increase in the temperature, suggesting a greater sorption feasibility at higher temperature. The *ΔH°* value was found to be 7.31 kJ/mol for La(III), 7.76 kJ/mol for Ce(III), 6.11 kJ/mol for Pr(III), and 8.45 kJ/mol for Nd(III) indicating the endothermic nature of sorption at 293–333 K. The positive value of *ΔS°* ranging from 25.39 to 33.79 kJ/mol·K suggested an increase in randomness or adsorbed species degree of freedom at the solid/aqueous solution interface in the sorption process. A plot of ln(*K_c_*) vs. 1/*T* was found to be linear with the high *R*^2^ values (0.934–0.985). Similar results were obtained for lignin (see Table S3). The calculated thermodynamic parameters revealed that the La(III), Ce(III), Pr(III) and Nd(III) ion sorption onto lignin had endothermic, spontaneous and more favourable character at higher temperatures.

Studying the influence of temperature on the La(III), Ce(III), Pr(III), and Nd(III) ions sorption process efficiency on the ALG_5_L_1_ composite, an increase in the equilibrium capacity values with the increasing temperature from 293 to 333 K was observed. In detail, the equilibrium capacities increased as follows: 105.05 to 109.56 mg/g for La(III), 93.83 to 97.97 mg/g for Ce(III), 92.99 to 97.98 mg/g for Pr(III) as well as 96.28 to 98.68 mg/g for Nd(III). This can be attributed to the increased kinetic energy of the molecules or pore diffusion rate. 

**Figure 6:**
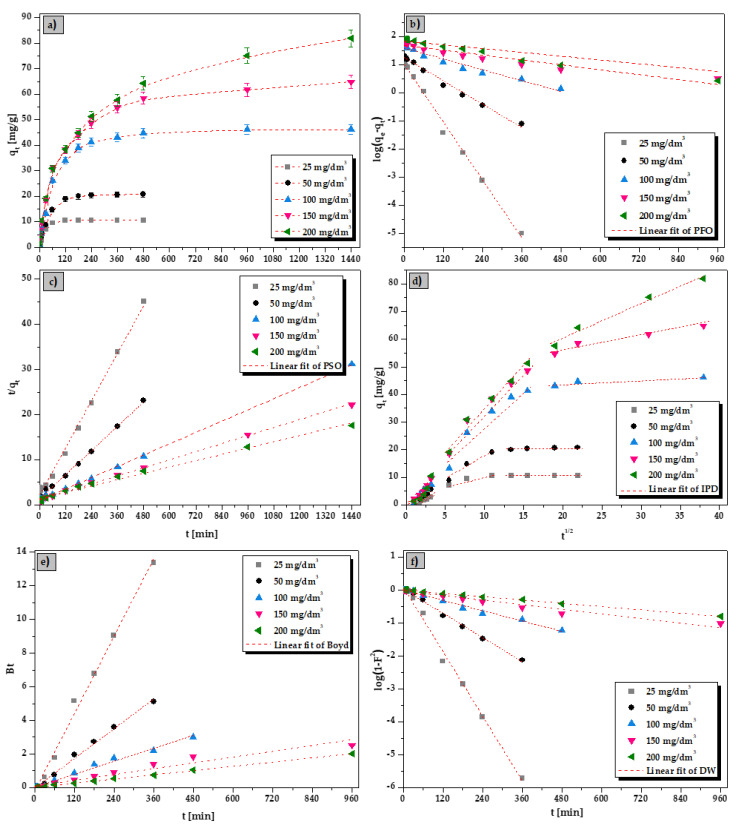
(**a**) Influence of interaction time and linear fitting of the kinetic models: (**b**) PFO, (**c**) PSO, (**d**) IPD, (**e**) Boyd, and (**f**) DW for the La(III) ions sorption on the ALG_5_L_1_ composite (conditions: *C*_0_ = 25–200 mg/dm^3^, *pH* = 5, *V* = 20 cm^3^, *m* = 0.05 g, *t* = 1–1440 min, *T* = 293 K).

The mathematical models used to describe the sorption kinetics included the pseudo-first order (PFO), the pseudo-second order (PSO), the intraparticle diffusion (IPD), the Boyd and the Dumwald–Wagner (DW) models. The La(III) ions sorption results from the kinetic studies are given in Table 1. The linear fitting of the kinetic models for La(III) ions sorption on the ALG_5_L_1_ composite is illustrated in Figure 6. On the other hand, the kinetic results for the Ce(III), Pr(III), and Nd(III) ions sorption are presented in the Supplementary Materials (Figures S4–S6). The all kinetic parameters presented in Table 1 (also Tables S4–S6) were determined from the slope and intercept of the straight lines in the plot of log(*q_e_*-*q_t_*) vs. *t* for the pseudo-first order kinetic model, *t*/*q_t_* vs. *t* for the pseudo-second order kinetic model, *q_t_* vs. *t*^1/2^ for the Weber-Morris intraparticle diffusion model, *Bt* vs. *t* for the Boyd model, and log(1-*F*^2^) vs. t for the Dumwald–Wagner model, respectively. 

**Table 1:** Calculated kinetic parameters for the La(III) ions sorption onto the ALG_5_L_1_ composite ^a^.

Kinetic Model	Equations	Parameter	ALG_5_L_1_				
		C_0_ [mg/dm^3^]	25	50	100	150	200
		***q_exp_*** **[mg/g]**	10.64	20.76	44.75	64.86	81.89
**PFO**	$\log(q_{1}-q_{t})=\log(q_{1})-\frac{k_{1}}{2.303}\times t$	***q*_1_** **[mg/g]**	10.21	17.61	37.99	48.42	68.67
		***k*_1_ × 10^−2^** **[1/min]**	3.94	1.59	0.74	0.34	0.26
		***R* ^2^**	0.995	0.991	0.970	0.912	0.963
**PSO**	$\frac{t}{q_{t}}=\frac{1}{k_{2}\times q_{2}^{2}}+\frac{1}{q_{2}}\times t$	***q*_2_** **[mg/g]**	11.42	22.35	48.03	67.10	85.75
	$h=k_{2}\times q_{2}^{2}$	***k*_2_ × 10^−2^ [g/mg∙min]**	0.36	0.15	0.042	0.021	0.010
		***h*** **[mg/g·min]**	0.47	0.76	0.95	0.96	0.72
		***R* ^2^**	0.996	0.998	0.999	0.998	0.992
**IPD**	$q_{t}=k_{i}\times t^{1/2}+C_{i}$	***k_i_*_1_** **[mg/g·min^1/2^]**	1.05	2.08	3.11	3.41	4.07
		***C*_1_**	0.07	0.18	0.55	0.94	1.04
		***R* ^2^**	0.959	0.975	0.974	0.932	0.867
		***k_i_*_2_** **[mg/g·min^1/2^]**	1.00	1.79	2.69	2.58	2.99
		***C*_2_**	0.64	1.35	2.15	8.17	4.01
		***R* ^2^**	0.883	0.982	0.933	0.964	0.978
		***k_i3_*** **[mg/g·min^1/2^]**	0.0007	0.092	0.25	0.48	1.24
		***C_3_***	10.63	18.84	37.51	46.67	35.60
		***R* ^2^**	0.599	0.857	0.751	0.955	0.982
**Boyd**	$\frac{q_{t}}{q_{e}}=1-\frac{6}{\pi^{2}}\exp(-Bt)$	***B***	13.38	5.10	3.00	2.53	1.99
	$F=\frac{q_{t}}{q_{e}}$	***R* ^2^**	0.996	0.995	0.988	0.999	0.999
**DW**	$\log(1-F^{2})=-\frac{K}{2.303t}$	***K***	0.038	0.014	0.006	0.003	0.002
		***R* ^2^**	0.996	0.997	0.991	0.959	0.999

^a^*k*_1_, *k*_2_ and *k_i_* are the rate constants of PFO [1/min], PSO [g/mg·min], and IPD [mg/g·min^1/2^] kinetic models, *q*_1_ and *q*_2_ are the adsorption capacities at equilibrium determined from the PFO and PSO kinetic models, *h* is the initial rate of adsorption [mg/g·min], *C_i_* is the boundary layer effect constant in the Weber-Morris equation, *B* is the rate coefficient [1/min], *K* is the adsorption constant rate [1/min], *F* is the sorbate fraction in the sorbent at a given time *t*: if 0 ≤ *F* ≤ 0.85 $Bt=2\pi -((\pi^{2}F)/3)-2\pi {\left(1-(\pi F)/3\right)}^{1/2}$ or if 0.86 ≤ *F* ≤ 1 $Bt=0.4977-\ln(1-\frac{q_{t}}{q_{e}})$.

Figure 6 (also Figures S4–S6) plot the kinetic profiles of La(III), Ce(III), Pr(III), and Nd(III) ions sorption using ALG_5_L_1_ as a sorbent. It can be clearly that the sorption process rate depends on the initial concentration of metal ions (Figure 6a). An increase in the initial concentration from 25 to 200 mg/dm^3^ resulted in the sorption process rate decrease and hence an increase in the time to establish the sorption equilibrium. For example, 50% La(III) ions sorption for the metal concentrations in the range of 25–200 mg/dm^3^ required the times ranging from 15 to 240 min, respectively. The Ce(III), Pr(III), and Nd(III) ions removal has a similar trend. Depending on the initial La(III), Ce(III), Pr(III), and Nd(III) solution concentrations, the sorption equilibrium state was established after 120 min for 25–50 mg/dm^3^, 480 min for 100 mg/dm^3^, and 1440 min for 150 and 200 mg/dm^3^. 

As follows from Table 1 (also Tables S4–S6), the correlation coefficients (*R*^2^) for the PFO model are not high in the whole La(III), Ce(III), Pr(III), and Nd(III) ions concentration range. Moreover, the evaluated *q*_1_ values calculated from the PFO equation differed from the experimental *q_exp_* values which means that the PFO kinetic model is not suitable to represent the sorption process. Considering the results obtained for the PSO model, the *R*^2^ values are large throughout the metal concentration range and tend towards unity (0.992–0.999). Moreover, the estimated *q*_2_ values calculated from the PSO equation were similar to the experimental *q_exp_* values. Additionally, the plots of the PSO kinetic model (Figure 6c and Figures S4–S6c) were rectilinear over the entire range and thus, the pseudo second-order model fits the data most satisfactorily. The initial rate of adsorption, *h*, determined for the pseudo-second order model increased when the increasing initial metal ions concentration from 25 to 150 mg/dm^3^ and after that decreased maybe due to the high competition of metal ions for the available active sites of composites which leads also to lower sorption rates.

As the pseudo-first and pseudo-second order kinetic models cannot identify the diffusion mechanism, the Weber-Morris intraparticle diffusion, the Boyd, and the Dumwald-Wagner kinetic models were applied in order to determine the participation of the diffusion process in the La(III), Ce(III), Pr(III), and Nd(III) ions sorption using the ALG_5_L_1_ composite. The Weber-Morris plot (Figure 6d) shows the multi-stages of sorption process as the fitted experimental data are not linear as well as the plot does not pass through the origin. This implies the existence of three stages that contribute to the sorption process and intraparticle diffusion is not a sole rate limiting step [30]. The first one is related to the adsorbate diffusion from the solution to the outer surface of the adsorbent. The next stage involves the slow adsorbate movement from the boundary layer to the sorbent surface (called film diffusion), and the third stage is related to the adsorbate movement to the adsorbent pore and after that adsorption equilibrium is achieved [31]. The calculated intraparticle diffusion values of *k_i_*_1_, *k_i_*_2_, *k_i3_* and *C*_1_, *C*_2_, *C_3_* differ enormously, and increase in the order *k_i_*_1_ > *k_i_*_2_ > *k_i3_*, and *C*_1_ > *C*_2_ > *C_3_*. Moreover, both *k_i_* and *C_i_* values increase with the increasing initial La(III), Ce(III), Pr(III), and Nd(III) concentrations, the higher the *C_i_* values, the greater the influence of the boundary layer on the sorption process. For the Boyd kinetic model, the plot intercept was not 0 but was close to 0 for all initial metal concentrations, it suggests that the predominant limiting step of sorption rate is intraparticle diffusion. The Dumwald–Wagner kinetic plots are linear with high correlation coefficients (*R*^2^ values were in the range 0.959–0.999) pointing out to the applicability of this kinetic model. The diffusion rate constants for the La(III) diffusion inside the ALG_5_L_1_ composite were 0.002–0.038 1/min and decrease with the increasing initial metal concentration. 

The sorption process kinetics of La(III), Ce(III), Pr(III) and Nd(III) ions was also determined for raw lignin (Figure S7 and Table S7). The sorption equilibrium state was achieved after 120 min. The kinetic parameters of the above metal ions on raw lignin were best described by the pseudo-second order kinetic model. This is confirmed by the correlation coefficient value close to unity (*R*^2^ = 0.999–1.000) obtained for all systems. In addition, the q_2_ values obtained from the pseudo-second order kinetic model calculations were close to the experimental data (*q_exp_*). In the case of k_2_ and h coefficient values, there was an increasing trend of these parameters in the following series: La(III) > Pr(III) > Nd(III) > Ce(III). 

The isotherms give insight into the maximum sorption capacities or the amount of sorbent needed to remove a given mass of metal ion from an aqueous system under the process conditions. The interpretation of the obtained isotherms through the determination of equilibrium parameters also provides valuable information on the nature of the interactions between the metal ions and the sorbent as well as the mechanism of the process taking place. 

**Figure 7:**
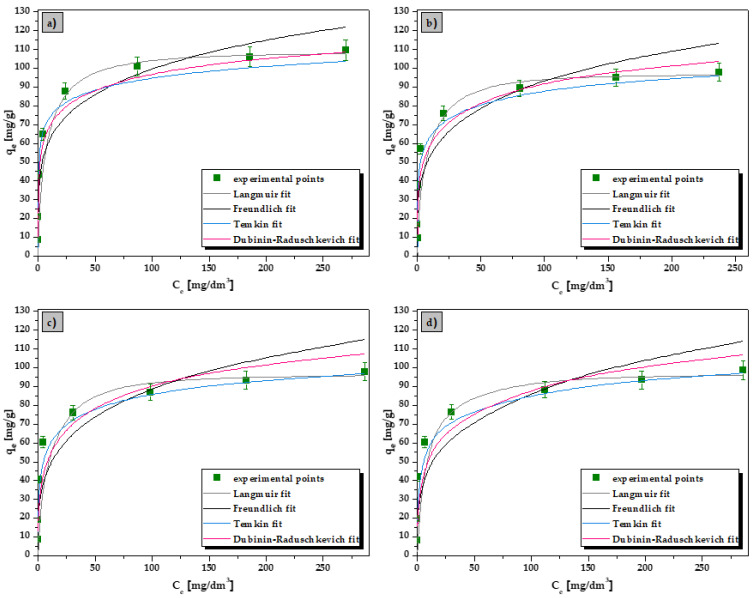
Nonlinear fitting of the isotherm models for (**a**) La(III), (**b**) Ce(III), (**c**) Pr(III), and (**d**) Nd(III) ions sorption on the ALG_5_L_1_ composite at 333 K (conditions: *C*_0_ = 25–500 mg/dm^3^, *pH* = 5, *V* = 20 cm^3^, *m* = 0.05 g, *t* = 1440 min).

In the sorption isotherm studies, the Langmuir, Freundlich, Temkin, and Dubinin–Radushkevich isotherms were employed to describe the equilibrium sorption process and to fit the experimental data. The isotherm parameters for the La(III) ions sorption are given in Table 2 (for the Ce(III), Pr(III) and Nd(III) ions sorption in the Supplementary Materials, Table S8). Moreover, the nonlinear fitting of isotherm models for La(III), Ce(III), Pr(III) and Nd(III) ions is presented in Figure 7.

**Table 2:** Calculated isotherm parameters for the La(III) ions sorption onto the ALG_5_L_1_ composite ^b^.

Isotherm Model	Equations	Parameters	ALG_5_L_1_		
		*T* [K]	293	313	333
		***q_exp_*** **[mg/g]**	105.05	107.95	109.56
**Langmuir**	$\frac{C_{e}}{q_{e}}=\frac{1}{K_{L}q_{m}}+\frac{C_{e}}{q_{m}}$	***q_m_*** **[mg/g]**	104.83	107.72	109.24
		***K_L_*** **[dm^3^/mg]**	0.19	0.25	0.28
		***R* ^2^**	0.996	0.998	0.998
		***χ* ^2^**	0.043	0.029	0.029
**Freundlich**	$\log q_{e}=\log K_{F}+\frac{1}{n}\log C_{e}$	***K_F_*** **[mg/g]**	39.97	43.14	43.56
		*n*	5.39	5.50	5.44
		***R* ^2^**	0.944	0.954	0.956
		***χ* ^2^**	0.682	1.134	1.286
**Temkin**	$q_{e}=(\frac{RT}{b_{T}})\ln A+(\frac{RT}{b_{T}})\ln C_{e}$	***A* × 10^3^** **[dm^3^/g]**	1.25	2.09	2.03
		***B*** **[J/mol]**	7.69	7.69	7.85
		***R* ^2^**	0.973	0.978	0.974
		***χ* ^2^**	0.472	0.338	0.338
**Dubinin–Raduschkevich**	$\ln q_{e}=\ln X_{m}-\beta \varepsilon^{2}$	***X_m_* × 10^−3^** **[mg/g]**	2.17	2.24	2.28
	$\varepsilon =RT\ln(1+\frac{1}{C_{e}})$	***Β* × 10^−3^** **[mol^2^/kJ^2^]**	1.25	1.20	1.20
		***E*** **[kJ/mol]**	19.98	20.44	20.41
		***R* ^2^**	0.963	0.979	0.977
		***χ* ^2^**	0.057	0.006	0.009

^b^*q_m_* is the maximum monolayer adsorption capacity [mg/g], *K_L_* is the Langmuir constant [dm^3^/mg], *K_F_* is the Freundlich constant, *n* is the heterogeneity factor, *b_T_* is the Temkin constant connected with the sorption heat [kJ/mol], *A* is the Temkin binding constant at equilibrium [dm^3^/g], *X_m_* is the Dubinin–Radushkevich constant related to the adsorption capacity [mg/g], *β* is the Dubinin–Radushkevich constant connected with the adsorption mean free energy [mol^2^/kJ^2^].

**Table 3:** EDX analysis results of ALG_5_L_1_ composite before and after the La(III) ions sorption.

	Element	Wt%	At%
Chemical composition of ALG_5_L_1_ composite before the La(III) ions sorption			
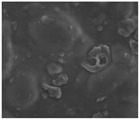	C K	43.03	52.39
	O K	48.76	44.59
	S K	0.17	0.08
	Cl K	0.09	0.04
	Ca K	7.96	2.91
	**Total**	**100.00**	**100.00**
Chemical composition of ALG_5_L_1_ composite after the La(III) ions sorption			
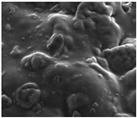	C K	42.27	55.28
	O K	43.60	42.77
	S K	0.17	0.08
	Ca K	1.00	0.40
	La L	12.96	1.47
	**Total**	**100.00**	**100.00**

It can be clearly seen from Figure 7 that the isotherm fitting of the La(III), Ce(III), Pr(III), and Nd(III) ions sorption on the ALG_5_L_1_ composite is the most compatible with the Langmuir isotherm compared with the Freundlich, Temkin and Dubinin–Raduschkevich ones. The correlation coefficients for the Langmuir isotherm fitting were 0.996, 0.998, 0.998 for the La(III) ions sorption at 293, 313 and 333 K, respectively (see Table 2). For the Ce(III), Pr(III), and Nd(III), the *R*^2^ values for the Langmuir isotherm were in the range of 0.997–0.998, 0.993–0.997 and 0.993–0.996, respectively (see Table S8). The obtained correlation coefficients close to 1 reveal that the monolayer chemical adsorption plays a main role in the sorption process of La(III), Ce(III), Pr(III) and Nd(III) ions on the ALG_5_L_1_ composite. Moreover, a good fit of a given isotherm model is indicated by the chi-square (*Χ*^2^) error value, which should be less than 1. Low *Χ*^2^ values were obtained for the Langmuir isotherm model (0.029–0.043 for La(III), 0.005–0.024 for Ce(III), 0.050–0.171 for Pr(III), and 0.073–0.157 for Nd(III)). The maximum monolayer adsorption capacity of ALG_5_L_1_ composite for La(III), Ce(III), Pr(III) and Nd(III) increased with the increasing temperature (from 293 to 333 K) and were 109.24, 97.6, 97.30 and 97.72 mg/g at 333 K, respectively. According to the *K_L_* parameter determined for the Langmuir model, its value increases with the increasing temperature indicating higher affinity of the sorbent for metal ions. Good fits were also obtained for the Temkin model which assumes that the entire adsorbent surface is not homogeneous from an energetic point of view [32]. The Dubinin–Radushkevich isotherm allowed for the determination of the parameter *E* defining the mean free energy, which made it possible to determine the nature of the sorption process-chemisorption or physisorption [33]. The values of parameter *E* for metal ions sorption on the ALG_5_L_1_ composite were in the range of 19.98–20.44 kJ/mol for La(III), 16.98–18.31 kJ/mol for Ce(III), 16.44–17.82 kJ/mol for Pr(III), and 15.90–18.57 kJ/mol for Nd(III) which indicates the chemical nature of the sorption process (surface complexation or ion exchange mechanism) [34]. Based on the results given in Table 2 (also Table S8), the adsorption capacity of the ALG_5_L_1_ composite towards the metal ions (La(III), Ce(III), Pr(III), and Nd(III)) is follows: La(III) > Nd(III) > Pr(III) ≈ Ce(III). Based on the *R*^2^, and *Χ*^2^ values, the best fitting isotherm adsorption order is: Langmuir > Temkin > Dubinin–Raduschkevich > Freundlich for the ALG_5_L_1_ composite. Moreover, according to the isotherm modelling data for the La(III), Ce(III), Pr(III), and Nd(III) ions sorption on raw lignin (see Table S9 and Figure S8) a similar fitting isotherm model to the above one was found. Considering the maximum monolayer adsorption capacities obtained for raw lignin (Table S9), the affinity series for the metal ions was as follows: Pr(III) (41.89 mg/g) > La(III) (39.08 mg/g) > Ce(III) (38.89 mg/g) > Nd(III) (31.00 mg/g). The above values of the maximum monolayer adsorption capacities for raw lignin were significantly smaller than those for the new synthetized calcium alginate–lignin composite one.

### 3.4. Comparison of Various Adsorbents for Rare Earth Elements Removal

In the literature, more and more adsorbents have been used to study their sorption potential towards the rare earth elements removal. The straw derived biochar was used for the sorption process of La(III) and Nd(III) ions. The maximum sorption capacities for La(III) and Nd(III) were 80.44 mg/g and 71.62 mg/g, respectively for the solution pH 5 at 293 K [35]. Kusrini et al. [36] used pectin extracted from durian rind for the La(III) ions sorption. The optimum sorption conditions were pH 4, temperature 298 K, and contact time 90 min for which 83.5% and 41.20 mg/g were obtained. Wang et al. [37] studied the adsorption behaviour of Nd(III) ions using o-carboxymethyl chitosan entrapped by silica. They reported that the adsorption capacity of o-carboxymethyl chitosan was significantly improved by support on SiO_2_ and reached 53.04 mg/g at 328 K. The montmorillonite was prepared, modified and applied to adsorb La(III) and Y(III) by Liu et al. [38]. The maximum adsorption capacities for the modified montmorillonite were 0.178 mmol/g for La(III) and 0.182 mmol/g for Y(III). In turn, Granados-Correa et al. [39] synthetized hydroxyapatite by the precipitation method and used as the adsorbent for La(III) and Eu(III) ions removal from aqueous solutions. The prepared hydroxyapatite revealed adsorption efficiences of 0.25 mg/g for La(III) and 0.94 mg/g for Eu(III). Wójcik [40] studied the sorption of light lanthanides(III), i.e., La(III), Ce(III), Pr(III) and Nd(III)) on Nitrolite and the sorption capacities of La(III), Ce(III), Pr(III) and Nd(III) were 4.77 mg/g, 4.45 mg/g, 4.30 mg/g, 4.13 mg/g, respectively at pH 9. Analyzing the sorption capacity values of the above adsorbents, it was found that the synthesized ALG_5_L_1_ composite exhibited a significant sorption potential towards the REE ions, the maximum sorption capacities at 333 K were equal to 109.56 mg/g for La(III), 97.97 mg/g for Ce(III), 97.98 mg/g for Pr(III), and 98.68 mg/g for Nd(III). In contrast to raw lignin the maximum sorption capacities at 333 K were 37.98 mg/g for La(III), 37.39 mg/g for Ce(III), 40.74 mg/g for Pr(III), and 30.06 mg/g for Nd(III). Therefore, it can be concluded that the calcium alginate–lignin composite is a promising biosorbent for the La(III), Ce(III), Pr(III), and Nd(III) ions removal from aqueous solutions.

### 3.5. Desorption and Reusability Studies

**Figure 8:**
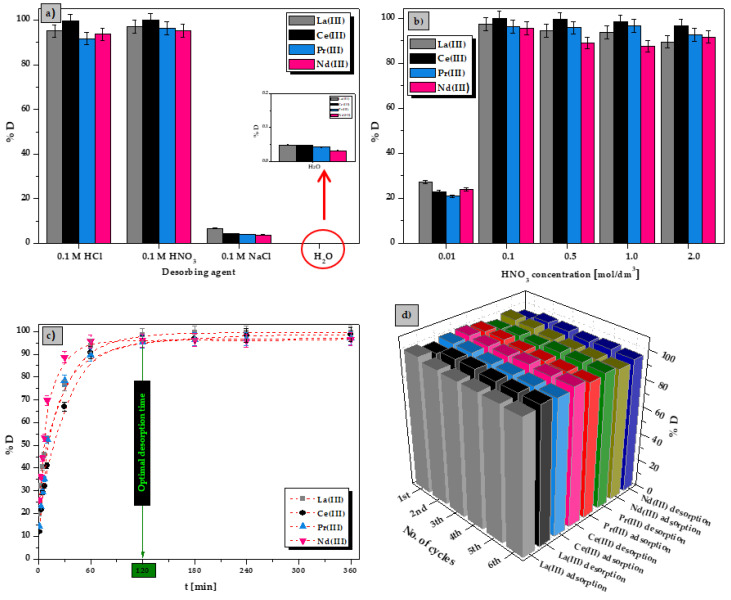
(**a**) Effect of desorbing agent on the La(III), Ce(III), Pr(III), and Nd(III) desorption efficiencies, (**b**) effect of HNO_3_ concentration on the desorption performance, (**c**) effect of desorbing time on the La(III), Ce(III), Pr(III), and Nd(III) desorption efficiencies, (**d**) reusability studies of ALG_5_L_1_ composite (conditions: *m* = 0.05 g, *V* = 20 cm^3^, *t* = 480 min, *T* = 293 K).

The desorption efficiency of La(III), Ce(III), Pr(III), and Nd(III) ions from the ALG_5_L_1_ composite was calculated as the ratio between the metal ions desorbed amount and the metal ions adsorbed one. First, the best desorbent agent was selected. For this purpose, four desorbing agents were used for the study: 0.1 M HCl, 0.1 M HNO_3_, 0.1 M NaCl and H_2_O. As illustrated in Figure 8a, in the case of HCl and HNO_3_, desorption efficiencies for La(III), Ce(III), Pr(III), and Nd(III) ions were very similar at around 93–99%. In contrast, NaCl could desorb maximum only 7% of the above metal ions. Distilled water was not an effective desorbing agent *(%D* below 0.1%). So HNO_3_ was chosen as the best desorbing agent for metal ions. The HNO_3_ concentrations of 0.01, 0.1, 0.5, 1.0, and 2.0 M were tested for elution of adsorbed La(III), Ce(III), Pr(III), and Nd(III) ions. It can be seen in Figure 8b that the desorption efficiency for the above metal ions initially increased with the increasing HNO_3_ concentration. However, insignificant changes of desorption efficiency were observed at the HNO_3_ concentrations over 0.1 M. Only for the desorption of Nd(III) ions, a slight decrease in *%D* was observed with the increasing HNO_3_ concentration from 0.5 to 2 M. Therefore, 0.1 M HNO_3_ with over 95% desorption efficiency was chosen as the optimal desorbing agent in terms of economy and efficiency. Moreover, the desorption time was a very significant parameter in batch processes. Figure 8c presents that the desorption efficiency for La(III), Ce(III), Pr(III), and Nd(III) ions increased with time. The desorption process was completed within 120 min.

Regeneration aspect of the sorption process is very essential in terms of its cost-effectiveness development by recycling the sorbent for example in the multiple cycles reuse studies. In order to scrutinize regeneration abilities for the ALG_5_L_1_ composite, consecutive adsorption-desorption cycles were repeated six times using the same sorbent. As shown in Figure 8d, the removal efficiencies of La(III), Ce(III), Pr(III), and Nd(III) ions for the recycled ALG_5_L_1_ composite were maintained at 95% even in the 6th cycle. Reusability studies proved that the new synthetized calcium alginate–lignin composite is a highly renewable sorbent.

## 4. Conclusions

A novel calcium alginate–lignin composite has been prepared by external gelation with calcium chloride at various ALG:L ratios (5:1, 2:1, 1:1, 1:2 and 1:5). ATR/FT-IR spectroscopy confirmed the lignin coating on the alginate surface. The morphology and surface properties of the composite were successfully described by the SEM, EDX, OM, AFM, BET, and XRD analyses. These composites were subsequently investigated as sorbents for La(III), Ce(III), Pr(III) and Nd(III) ions from aqueous solutions. Among the five types of ALG:L composites those with the ALG:L ratio of 5:1 (ALG_5_L_1_) showed the greatest adsorption capacities for La(III), Ce(III), Pr(III), and Nd(III) ions, and thus were used for further sorption studies. The experiment results revealed that lignin encapsulation in the alginate increased over 2.5-times the maximum sorption capacities of raw lignin. The optimum sorption conditions of the ALG_5_L_1_ composite were established at solution pH 5, mass 0.05 g, time 1440 min, and temperature 333 K. The existence of Fe(III) ions during the La(III), Ce(III), Pr(III), and Nd(III) ions sorption on the ALG_5_L_1_ composite had a significant effect (above 50% sorption efficiency decrease). The kinetic studies revealed that the sorption process of La(III), Ce(III), Pr(III), and Nd(III) ions on the ALG_5_L_1_ composite follows the pseudo-second order equation and the intraparticle diffusion is one of the main rate limiting steps in the sorption process. The Langmuir equation fits the isotherm equilibrium data of four elements more than the Freundlich, Temkin, and Dubinin–Raduschkevich equations (*R*^2^ > 0.993 and *Χ*^2^ < 0.157). More than 95% of adsorbed La(III), Ce(III), Pr(III), and Nd(III) ions were desorbed using 0.1 M HNO_3_ after 120 min contact time. After six sorption/desorption cycles no noticeable efficiency decrease is observed. The results of this study showed that the new calcium alginate–lignin composite is characterized by high stability, reusability and high adsorption capacity for REEs, therefore, it proves to be a very promising sorbent. 

## Data Availability

Data is contained within the article and supplementary material.

## Supplementary Materials

The following are available online at https://www.mdpi.com/article/10.3390/ma15030944/s1. Figure S1: Sorption effectiveness results depending on the ALG:L ratios (conditions: *C*_0_ = 100 mg/dm^3^, *pH* = 5, *m* = 0.05 g, *V* = 20 cm^3^, *t* = 480 min, *T* = 293 K). Figure S2: XRD patterns of calcium alginate, lignin, and calcium-alginate lignin composite. Figure S3: Influence of composite mass on La(III), Ce(III), Pr(III), and Nd(III) ions sorption by the ALG_5_L_1_ composite (conditions: *C*_0_ = 100 mg/dm^3^, *pH* = 5, *m* = 0.05–0.5 g, *V* = 20 cm^3^, *t* = 480 min, *T*= 293 K). Figure S4: (a) Influence of interaction time and linear fitting of kinetic models: (b) PFO, (c) PSO, (d) IPD, (e) Boyd, and (f) DW for Ce(III) ions sorption on the ALG_5_L_1_ composite. Figure S5: (a) Influence of interaction time and linear fitting of kinetic models: (b) PFO, (c) PSO, (d) IPD, (e) Boyd, and (f) DW for Pr(III) ions sorption on the ALG_5_L_1_ composite. Figure S6: (a) Influence of interaction time and linear fitting of kinetic models: (b) PFO, (c) PSO, (d) IPD, (e) Boyd, and (f) DW for Nd(III) ions sorption on the ALG_5_L_1_ composite. Figure S7: (a) Influence of interaction time and linear fitting of kinetic models: (b) PFO, (c) PSO, (d) IPD, (e) Boyd, and (f) DW for La(III), Ce(III), Pr(III), and Nd(III) ions sorption onto lignin for *C*_0_ = 100 mg/dm^3^. Figure S8: Nonlinear fitting of isotherm models for (a) La(III), (b) Ce(III), (c) Pr(III), and (d) Nd(III) ions sorption on lignin at 333 K. Table S1: Textural parameters of the studied biosorbents. Table S2: Calculated thermodynamic parameters for the La(III), Ce(III), Pr(III), and Nd(III) ions sorption onto ALG_5_L_1_ composite. Table S3: Calculated thermodynamic parameters for La(III), Ce(III), Pr(III), and Nd(III) ions sorption onto lignin. Table S4: Calculated kinetic parameters for Ce(III) ions sorption onto the ALG_5_L_1_ composite. Table S5: Calculated kinetic parameters for Pr(III) ions sorption onto the ALG_5_L_1_ composite. Table S6: Calculated kinetic parameters for Nd(III) ions sorption onto the ALG_5_L_1_ composite. Table S7: Calculated kinetic parameters for La(III), Ce(III), Pr(III), and Nd(III) ions sorption onto lignin for *C*_0_ = 100 mg/dm^3^. Table S8: Calculated isotherm parameters for Ce(III), Pr(III), and Nd(III) ions sorption onto the ALG_5_L_1_ composite. Table S9: Calculated isotherm parameters for La(III), Ce(III), Pr(III), and Nd(III) ions sorption onto lignin.
