# Supplementary material for: Fabrication, Characterization and Evaluation of an Alginate–Lignin Composite for Rare-Earth Elements Recovery
Source: Materials (Basel). 2022 Jan 26;15(3):944. doi: 10.3390/ma15030944 (PMC8838038; doi:10.3390/ma15030944)
Supplement: Supplementary file 1 [file materials-15-00944-s001.zip › materials-1558450-supplementary.pdf]

materials

MDPI

Supplementary materials

Fabrication, Characterization and Evaluation of an Alginate-Lignin Composite for Rare-Earth Elements Recovery

Dominika Fila, Zbigniew Hubicki and Dorota Kołodyńska

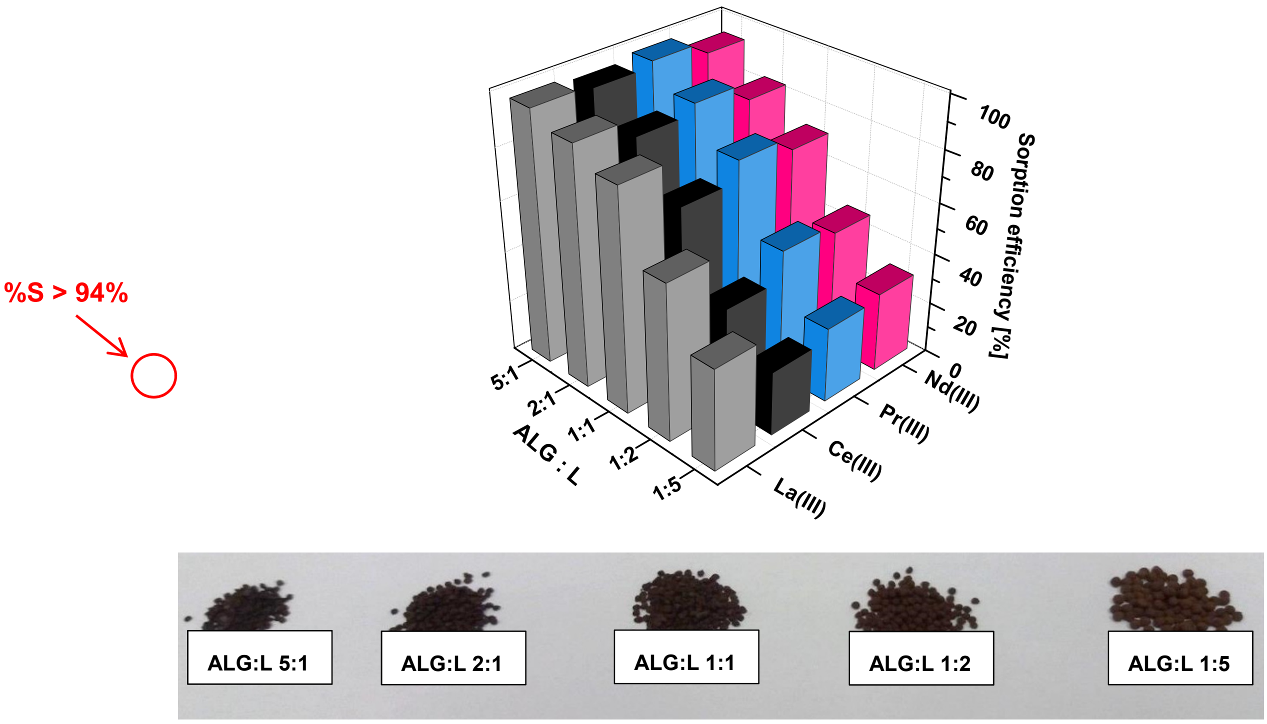

Figure S1. Sorption effectiveness results depending on the ALG:L ratios (conditions: C_0 = 100 mg/dm^3, ***pH*** = 5, ***m*** = 0.05 g, ***V*** = 20 cm^3, ***t*** = 480 min, ***T*** = 293 K).

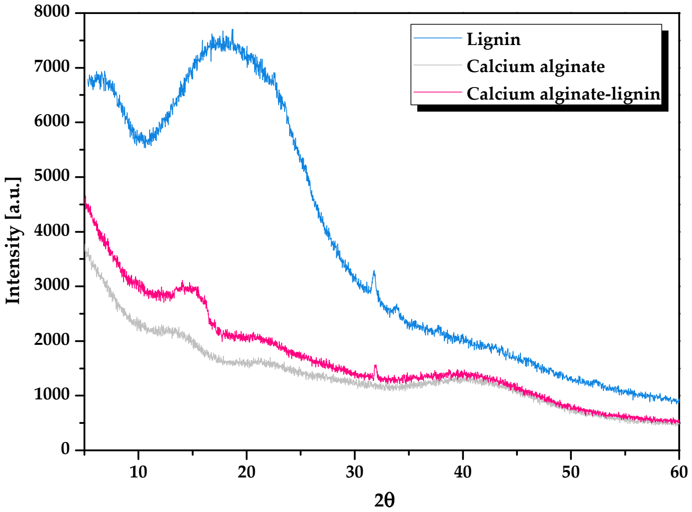

Figure S2. XRD patterns of calcium alginate, lignin, and calcium alginate-lignin composite.

Table S1. Textural parameters of the studied biosorbents.

Biosorbent		*Textural parameters*		
		S_{BET} [m²/g]	D [nm]	V_{total} [cm³/g]
Calcium alginate	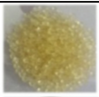	4.707	3.144	0.773×10^{-2}
Lignin	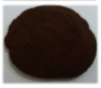	1.918	6.107	$0.385\ 10^{-2}$
Calcium alginate-lignin (ALG_5L_1)	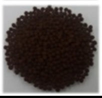	8.018	2.975	1.070×10^{-2}

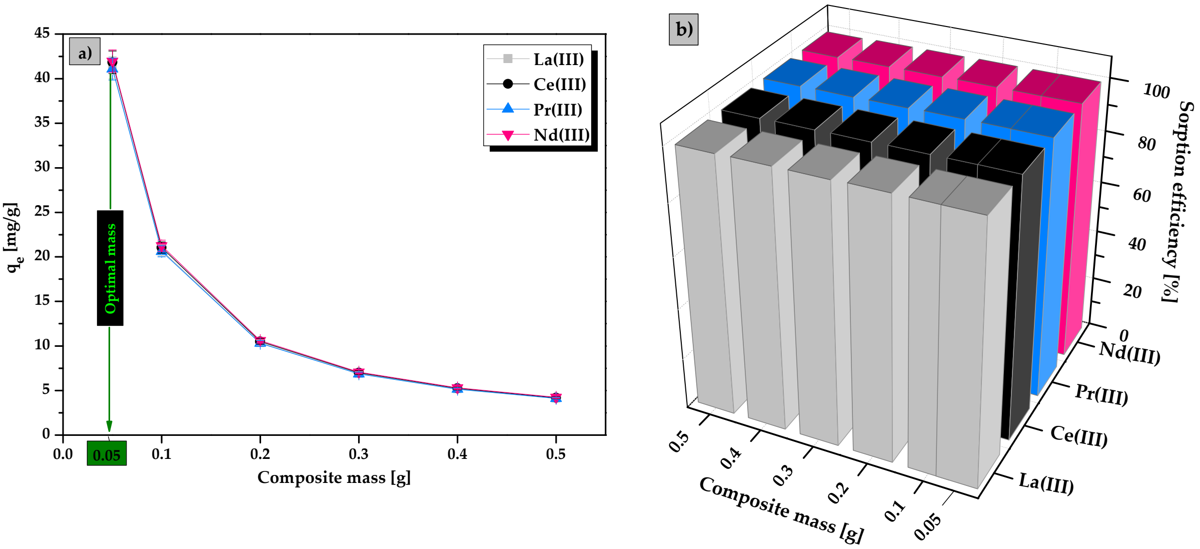

Figure S3. Influence of composite mass on La(III), Ce(III), Pr(III), and Nd(III) ions sorption by the ALG_5L_1 composite (conditions: C_0 = 100 mg/dm³, pH = 5, m = 0.05-0.5 g, V = 20 cm³, t = 480 min, T = 293 K).

Table S2. Calculated thermodynamic parameters for the La(III), Ce(III), Pr(III), and Nd(III) ions sorption onto ALG_5L_1 composite.

ALG_5L_1 + sorbed metal ions	$\Delta H°$ [kJ/mol]	$\Delta S°$ [J/mol·K]	R^2	$\Delta G°$ [kJ/mol]		
				293 K	313 K	333 K
**ALG_5L_1 + La(III)**	7.31	32.27	0.985	-18.94	-20.83	-22.52
**ALG_5L_1 + Ce(III)**	7.76	33.79	0.973	-18.93	-20.89	-22.56
**ALG_5L_1 + Pr(III)**	6.11	25.39	0.974	-18.19	-19.74	-21.51
**ALG_5L_1 + Nd(III)**	8.45	32.20	0.934	-17.89	-19.45	-21.50

Table S3. Calculated thermodynamic parameters for La(III), Ce(III), Pr(III), and Nd(III) ions sorption onto lignin.

Lignin + sorbed metal ions	$\Delta H°$ [kJ/mol]	$\Delta S°$ [J/mol·K]	R^2	$\Delta G°$ [kJ/mol]		
				293 K	313 K	333 K
**Lignin + La(III)**	3.98	1.03	1.000	-13.15	-14.32	-15.49
**Lignin + Ce(III)**	6.79	7.70	0.995	-12.30	-13.54	-14.90
**Lignin + Pr(III)**	9.27	18.20	0.998	-12.91	-14.38	-15.93
**Lignin + Nd(III)**	4.65	0.71	0.999	-11.98	-13.10	-14.25

Table S4. Calculated kinetic parameters for Ce(III) ions sorption onto the ALG_5L_1 composite.

Kinetic model	Equations	Parameter	ALG_5L_1				
		C_0 [mg/dm³]	25	50	100	150	200
		q_{exp} [mg/g]	9.56	19.85	40.65	61.04	86.13
PFO	$\log(q_1-q_t)=\log(q_1)-\frac{k_1}{2.303}\times t$	q_1 [mg/g]	5.50	17.08	38.62	48.57	76.94
		$k_1\times10^{-2}$ [1/min]	2.15	2.18	1.02	0.37	0.26
		R^2	0.883	0.967	0.996	0.936	0.985
PSO	$\frac{t}{q_t}=\frac{1}{k_2\times q_2^2}+\frac{1}{q_2}\times t$	q_2 [mg/g]	10.14	23.20	42.88	65.88	91.62
	$h=k_2\times q_2^2$	$k_2\times10^{-2}$ [g/mg·min]	0.49	0.076	0.045	0.014	0.007
		h [mg/g·min]	0.50	0.41	0.83	0.61	0.62
		R^2	0.997	0.997	0.998	0.992	0.984
IPD	$q_t=k_i\times t^{1/2}+C_i$	k_{i1} [mg/g·min$^{1/2}$]	0.92	1.59	2.05	3.28	1.80
		C_1	0.45	0.68	1.18	3.30	0.37
		R^2	0.974	0.953	0.986	0.988	0.904
		k_{i2} [mg/g·min$^{1/2}$]	0.89	1.96	2.50	2.78	3.33
		C_2	0.70	1.30	0.85	1.73	3.15
		R^2	0.859	0.955	0.980	0.981	0.996
		k_{i3} [mg/g·min$^{1/2}$]	0.003	0.013	0.17	0.45	1.41
		C_3	9.49	19.57	35.16	44.38	33.63
		R^2	0.978	0.939	0.568	0.925	0.980
Boyd	$\frac{q_t}{q_e}=1-\frac{6}{\pi^2}\exp(-Bt)$	B	6.24	6.75	4.52	2.79	1.98
	$F=\frac{q_t}{q_e}$	R^2	0.891	0.971	0.992	0.873	0.997
DW	$\log(1-F^2)=-\frac{K}{2.303t}$	K	0.020	0.020	0.009	0.003	0.002
		R^2	0.896	0.973	0.990	0.972	0.997

Table S5. Calculated kinetic parameters for Pr(III) ions sorption onto the ALG_5L_1 composite.

Kinetic model	Equations	Parameter	ALG_5L_1				
		C_0 [mg/dm³]	25	50	100	150	200
		q_{exp} [mg/g]	9.15	19.10	39.33	60.06	74.65
PFO	$\log(q_1 - q_t) = \log(q_1) - \frac{k_1}{2.303} \times t$	q_1 [mg/g]	5.25	17.06	39.37	50.67	63.91
		$k_1 \times 10^{-2}$ [1/min]	2.46	2.36	1.05	0.39	0.28
		R^2	0.899	0.987	0.995	0.965	0.978
PSO	$\frac{t}{q_t} = \frac{1}{k_2 \times q_2^2} + \frac{1}{q_2} \times t$	q_2 [mg/g]	9.64	20.84	42.22	64.86	79.26
	$h = k_2 \times q_2^2$	$k_2 \times 10^{-2}$ [g/mg·min]	0.58	0.15	0.035	0.014	0.010
		h [mg/g·min]	0.54	0.66	0.62	0.59	0.62
		R^2	0.998	0.995	0.996	0.996	0.991
IPD	$q_t = k_i \times t^{1/2} + C_i$	k_{i1} [mg/g·min$^{1/2}$]	1.15	1.63	1.24	2.16	4.03
		C_1	0.80	1.11	0.51	1.66	4.09
		R^2	0.970	0.901	0.993	0.821	0.912
		k_{i2} [mg/g·min$^{1/2}$]	0.80	1.78	2.24	2.57	2.75
		C_2	1.13	0.27	2.14	2.26	2.78
		R^2	0.871	0.953	0.995	0.988	0.981
		k_{i3} [mg/g·min$^{1/2}$]	0.001	0.015	0.21	0.49	1.06
		C_3	9.12	18.79	32.88	42.42	35.53
		R^2	0.974	0.735	0.482	0.858	0.975
Boyd	$\frac{q_t}{q_e} = 1 - \frac{6}{\pi^2} \exp(-Bt)$	B	7.10	7.46	4.40	3.05	2.21
	$F = \frac{q_t}{q_e}$	R^2	0.905	0.989	0.985	0.913	0.998
DW	$\log(1 - F^2) = -\frac{K}{2.303t}$	K	0.023	0.022	0.009	0.003	0.002
		R^2	0.909	0.990	0.982	0.985	0.998

Table S6. Calculated kinetic parameters for Nd(III) ions sorption onto the ALG_5L_1 composite.

Kinetic model	Equations	Parameter	ALG_5L_1				
		C_0 [mg/dm³]	25	50	100	150	200
		q_{exp} [mg/g]	8.43	20.13	42.62	65.71	78.16
PFO	$\log(q_1 - q_t) = \log(q_1) - \frac{k_1}{2.303} \times t$	q_1 [mg/g]	6.20	16.75	41.06	51.20	64.59
		$k_1 \times 10^{-2}$ [1/min]	2.22	2.12	1.00	0.35	0.26
		R^2	0.954	0.955	0.978	0.938	0.966
PSO	$\frac{t}{q_t} = \frac{1}{k_2 \times q_2^2} + \frac{1}{q_2} \times t$	q_2 [mg/g]	9.01	22.59	44.23	68.38	80.20
	$h = k_2 \times q_2^2$	$k_2 \times 10^{-2}$ [g/mg·min]	0.46	0.11	0.058	0.019	0.013
		h [mg/g·min]	0.38	0.54	1.13	0.90	0.81
		R^2	0.997	0.992	0.997	0.997	0.986
IPD	$q_t = k_i \times t^{1/2} + C_i$	k_{i1} [mg/g·min$^{1/2}$]	0.87	1.65	2.40	2.29	3.89
		C_1	0.58	1.40	1.63	0.34	1.38
		R^2	0.929	0.986	0.880	0.797	0.939
		k_{i2} [mg/g·min$^{1/2}$]	0.74	2.00	2.10	2.53	2.80
		C_2	0.71	1.47	4.68	7.98	4.02
		R^2	0.910	0.931	0.983	0.971	0.985
		k_{i3} [mg/g·min$^{1/2}$]	0.005	0.016	0.31	0.52	1.09
		C_3	8.32	19.79	32.88	46.53	37.21
		R^2	0.709	0.745	0.472	0.889	0.977
Boyd	$\frac{q_t}{q_e} = 1 - \frac{6}{\pi^2} \exp(-Bt)$	B	6.62	6.21	4.43	2.70	1.96
	$F = \frac{q_t}{q_e}$	R^2	0.960	0.959	0.959	0.876	0.994
DW	$\log(1 - F^2) = -\frac{K}{2.303t}$	K	0.020	0.019	0.009	0.003	0.002
		R^2	0.962	0.960	0.955	0.971	0.995

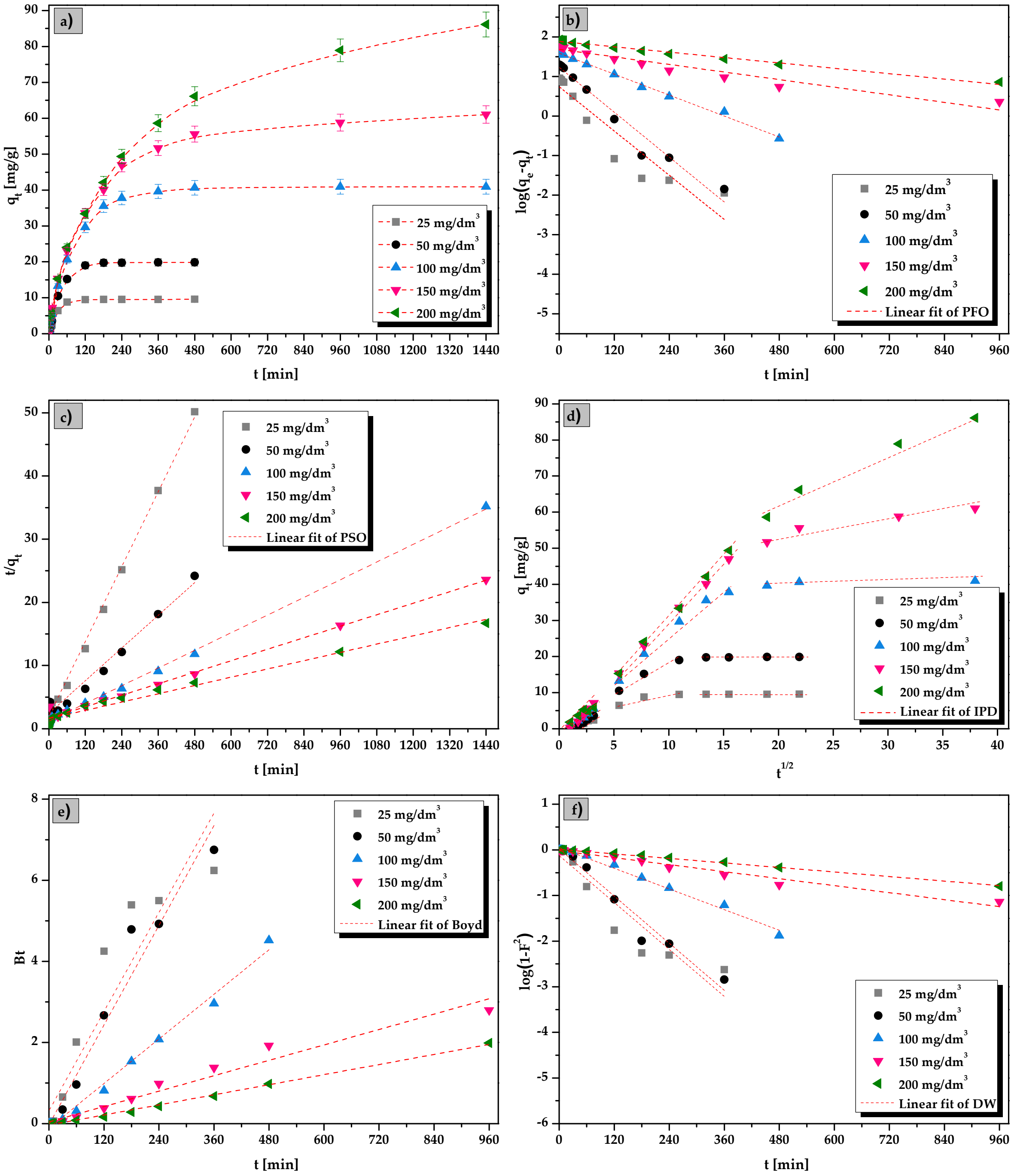

Figure S4. (**a**) Influence of interaction time and linear fitting of kinetic models: (**b**) PFO, (**c**) PSO, (**d**) IPD, (**e**) Boyd, and (**f**) DW for Ce(III) ions sorption on the ALG_5L_1 composite.

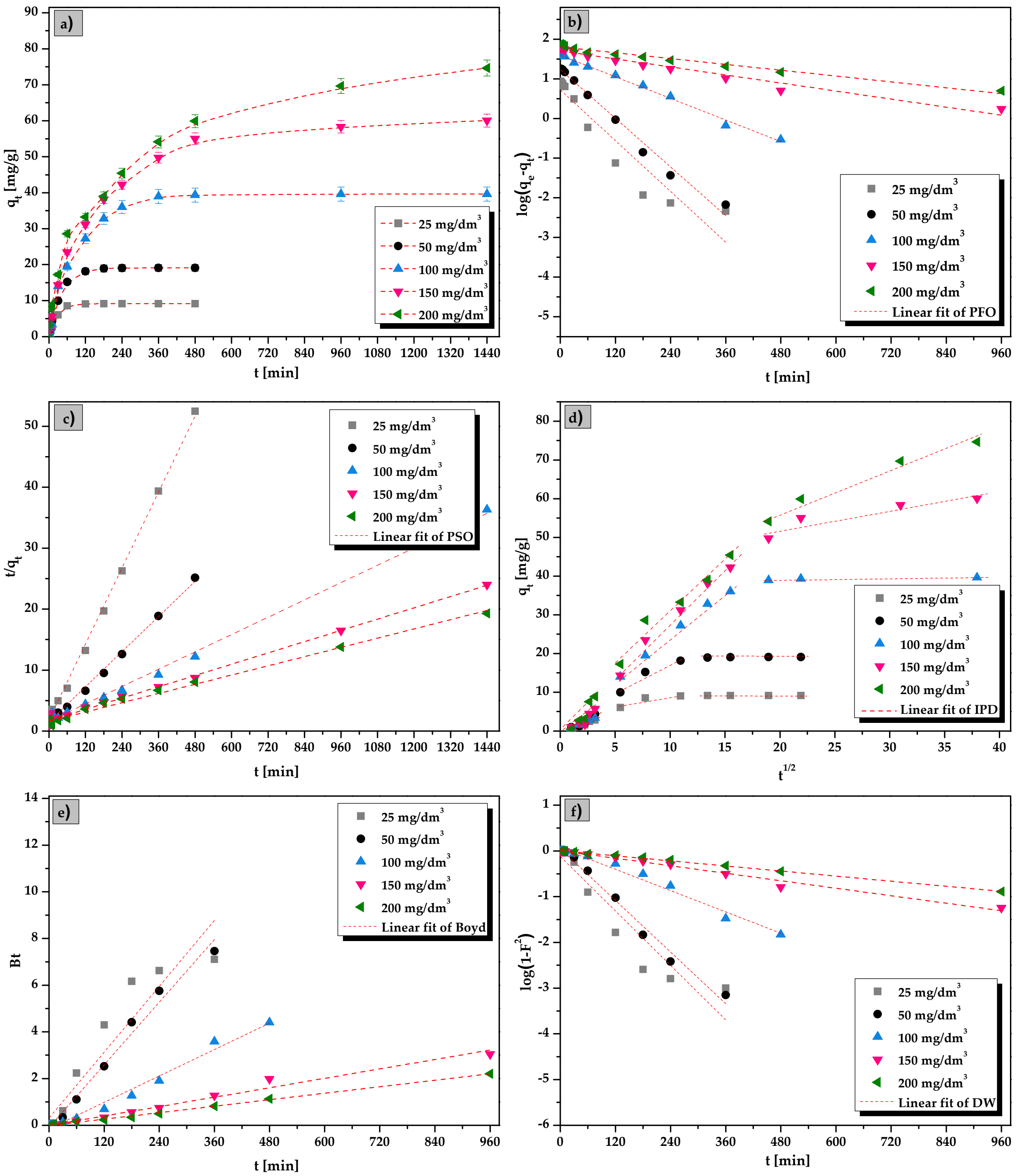

Figure S5. (**a**) Influence of interaction time and linear fitting of kinetic models: (**b**) PFO, (**c**) PSO, (**d**) IPD, (**e**) Boyd, and (**f**) DW for Pr(III) ions sorption on the ALG5L1 composite.

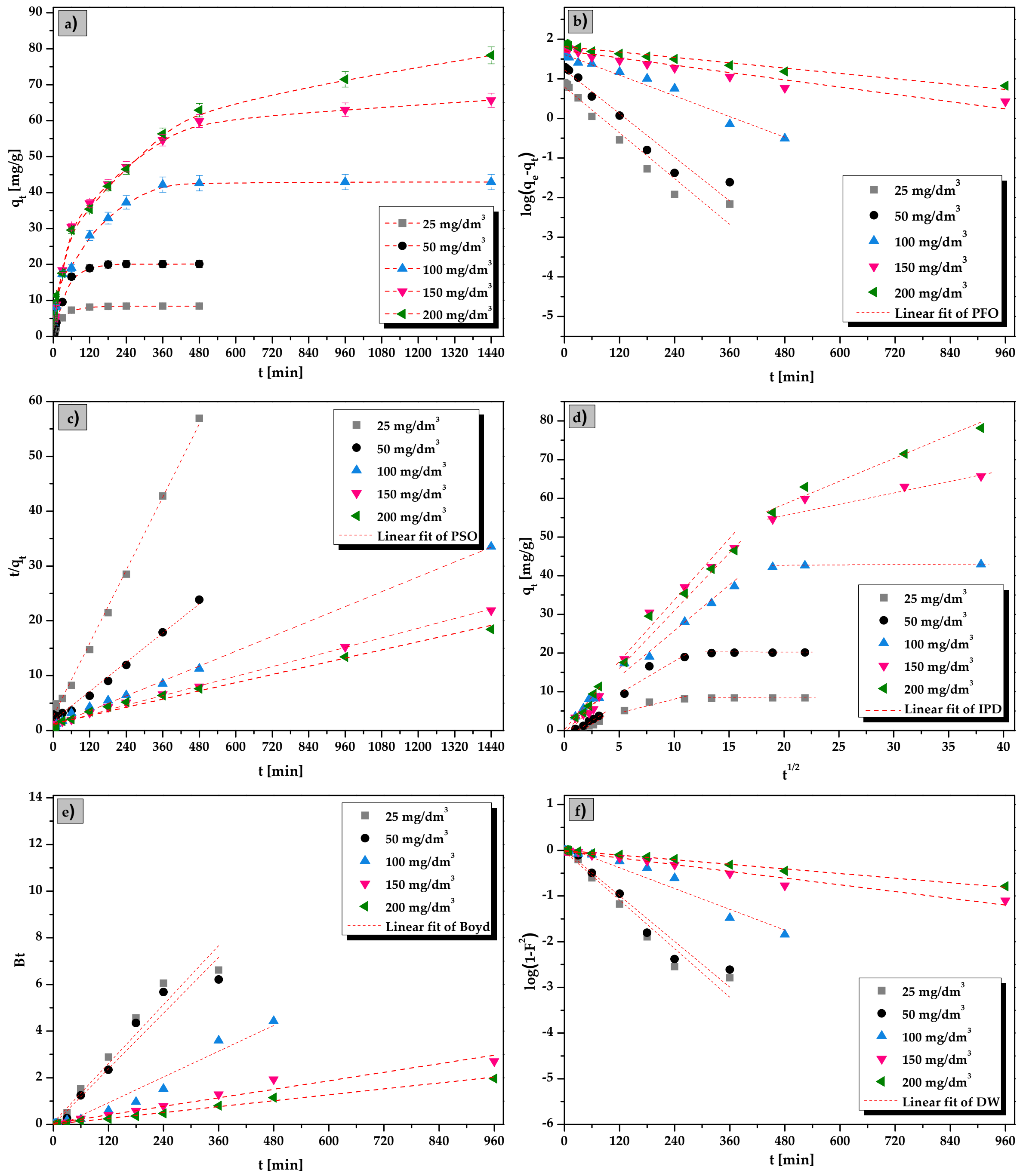

Figure S6. (**a**) Influence of interaction time and linear fitting of kinetic models: (**b**) PFO, (**c**) PSO, (**d**) IPD, (**e**) Boyd, and (**f**) DW for Nd(III) ions sorption on the ALG_5L_1 composite.

Table S7. Calculated kinetic parameters for La(III), Ce(III), Pr(III), and Nd(III) ions sorption onto raw lignin for C_0 =100 mg/dm[3].

Kinetic model	Equations	Parameter / Ions	Lignin La(III)	Ce(III)	Pr(III)	Nd(III)
		q_{exp} [mg/g]	22.72	23.66	20.27	18.85
**PFO**	$\log(q_1 - q_t) = \log(q_1) - \frac{k_1}{2.303} \times t$	q_1 [mg/g]	7.87	13.05	7.74	7.11
		k_1×10^{-2} [1/min]	1.60	1.87	1.98	1.38
		R^2	0.946	0.957	0.899	0.962
**PSO**	$\frac{t}{q_t} = \frac{1}{k_2 \times q_2^2} + \frac{1}{q_2} \times t$	q_2 [mg/g]	22.83	24.03	20.49	18.93
	$h = k_2 \times q_2^2$	k_2×10^{-2} [g/mg·min]	1.25	0.60	1.20	1.11
		h [mg/g·min]	5.97	3.46	5.04	4.00
		R^2	0.999	0.999	1.000	0.999
**IPD**	$q_t = k_i \times t^{1/2} + C_i$	k_{i1} [mg/g·min$^{1/2}$]	4.10	3.56	4.33	2.53
		C_1	5.37	2.76	1.24	5.86
		R^2	0.937	0.955	0.963	0.986
		k_{i2} [mg/g·min$^{1/2}$]	0.47	0.95	0.65	0.54
		C_2	16.57	12.53	13.42	12.01
		R^2	0.938	0.911	0.918	0.965
		k_{i3} [mg/g·min$^{1/2}$]	0.19	0.24	0.09	0.19
		C_3	19.30	19.42	18.75	15.49
		R^2	0.928	0.923	0.770	0.940
**Boyd**	$\frac{q_t}{q_e} = 1 - \frac{6}{\pi^2} \exp(-Bt)$	B	4.53	4.94	4.76	3.69
	$F = \frac{q_t}{q_e}$	R^2	0.956	0.963	0.911	0.972
**DW**	$\log(1 - F^2) = -\frac{K}{2.303t}$	K	0.015	0.017	0.019	0.013
		R^2	0.960	0.960	0.917	0.976

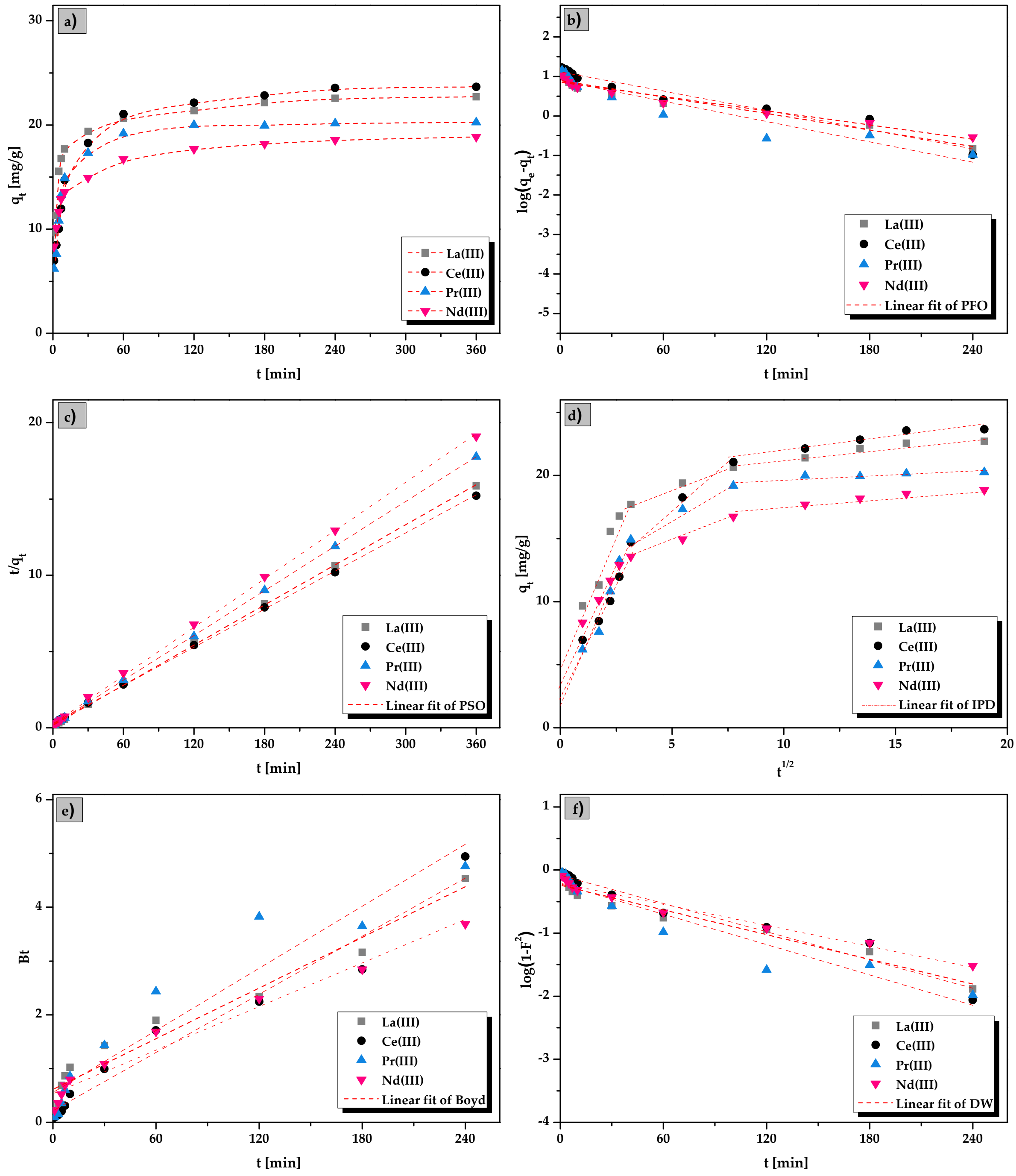

Figure S7. (**a**) Influence of interaction time and linear fitting of kinetic models: (**b**) PFO, (**c**) PSO, (**d**) IPD, (**e**) Boyd, and (**f**) DW for La(III), Ce(III), Pr(III), and Nd(III) ions sorption onto raw lignin for C_0 =100 mg/dm^3.

Table S8. Calculated isotherm parameters for Ce(III), Pr(III), and Nd(III) ions sorption onto the ALG_5L_1 composite.

**Isotherm model**	**Parameter**	**Ce(III)**			**Pr(III)**			**Nd(III)**		
	***T* [K]**	**293**	**313**	**333**	**293**	**313**	**333**	**293**	**313**	**333**
	**q_{exp} [mg/g]**	93.83	96.58	97.97	92.99	97.85	97.98	96.28	98.46	98.68
**Langmuir**	**q_m [mg/g]**	94.56	96.68	97.66	92.02	95.71	97.30	94.89	97.43	97.72
	**K_L [dm^3/mg]**	0.27	0.32	0.33	0.15	0.17	0.22	0.13	0.16	0.20
	**R^2**	0.998	0.997	0.998	0.995	0.993	0.997	0.993	0.994	0.996
	**χ^2**	0.005	0.013	0.024	0.099	0.171	0.050	0.157	0.101	0.073
**Freundlich**	**K_F [mg/g]**	31.26	34.36	36.54	30.47	29.93	31.61	31.40	28.54	28.75
	**n**	4.43	4.68	4.83	4.74	4.34	4.38	4.88	4.18	4.10
	**R^2**	0.876	0.864	0.945	0.934	0.840	0.864	0.965	0.829	0.823
	**χ^2**	1.973	1.847	2.102	0.724	1.400	2.541	0.169	1.317	2.071
**Temkin**	**$A \times 10^2$ [dm^3/g]**	0.89	1.70	3.30	1.82	0.76	0.99	3.02	0.52	0.48
	**B [J/mol]**	9.380	9.036	8.526	8.022	9.423	9.441	7.661	9.885	10.203
	**R^2**	0.977	0.968	0.991	0.984	0.974	0.985	0.961	0.969	0.976
	**χ^2**	0.003	0.004	0.036	0.348	0.148	0.012	0.938	0.122	0.022
**Dubinin-Radusch-kevich**	**$X_m \times 10^{-3}$ [mg/g]**	2.42	2.36	2.37	2.11	2.52	2.58	2.00	2.57	2.67
	**$\beta \times 10^{-3}$ [mol^2/kJ^2]**	1.73	1.58	1.49	1.57	1.85	1.80	1.45	1.94	1.98
	**E [kJ/mol]**	16.98	17.78	18.31	17.82	16.44	16.66	18.57	16.07	15.90
	**R^2**	0.898	0.880	0.981	0.963	0.884	0.911	0.978	0.863	0.858
	**χ^2**	0.420	0.237	0.310	0.016	0.331	0.833	0.354	0.262	0.613

Table S9. Calculated isotherm parameters for La(III), Ce(III), Pr(III), and Nd(III) ions sorption onto raw lignin.

**Isotherm model**	**Parameter**	**La(III)**			**Ce(III)**			**Pr(III)**			**Nd(III)**		
	***T* [K]**	**293**	**313**	**333**	**293**	**313**	**333**	**293**	**313**	**333**	**293**	**313**	**333**
	q_{exp} **[mg/g]**	34.69	36.25	37.98	26.41	30.08	37.39	32.22	36.90	40.74	24.07	27.57	30.06
**Langmuir**	q_m **[mg/g]**	35.93	37.24	39.08	27.06	30.84	38.89	33.15	37.85	41.89	24.91	28.34	31.00
	K_L **[dm³/mg]**	0.054	0.060	0.069	0.082	0.084	0.044	0.052	0.055	0.063	0.051	0.042	0.041
	R^2	0.997	0.996	0.997	0.999	0.999	0.997	0.996	0.996	0.999	0.999	0.995	0.994
	χ^2	0.003	0.007	0.002	0.001	0.001	0.004	0.013	0.018	0.008	0.002	0.020	0.029
**Freundlich**	K_F **[mg/g]**	10.01	10.14	11.31	8.62	9.54	7.77	6.98	7.30	6.83	5.15	5.97	6.39
	n	4.66	4.51	4.69	5.06	4.91	3.65	3.67	3.41	3.00	3.66	3.80	3.75
	R^2	0.977	0.989	0.989	0.942	0.960	0.942	0.953	0.951	0.904	0.956	0.988	0.986
	χ^2	0.070	0.104	0.154	0.231	0.291	0.343	0.306	0.534	1.410	0.346	0.117	1.286
**Temkin**	A **[dm³/g]**	6.30	9.55	15.10	8.03	8.61	1.72	2.69	2.32	1.49	1.66	2.11	2.19
	B **[J/mol]**	4.37	4.31	4.32	3.34	3.77	5.71	4.63	5.47	6.73	3.78	3.99	4.31
	R^2	0.947	0.952	0.956	0.973	0.990	0.987	0.991	0.995	0.987	0.978	0.991	0.990
	χ^2	0.005	0.014	0.005	0.040	0.039	0.005	0.001	0.001	0.069	0.028	0.007	0.337
**Dubinin-Radusch-kevich**	$X_m \times 10^{-3}$ **[mg/g]**	0.81	0.85	0.88	0.65	0.75	1.08	0.92	1.13	1.49	0.71	0.74	0.81
	$\beta \times 10^{-3}$ **[mol²/kJ²]**	1.95	1.94	1.83	1.95	1.96	2.74	2.61	2.79	3.27	2.74	2.55	2.57
	E **[kJ/mol]**	16.01	16.07	16.54	15.99	15.96	13.51	13.84	13.38	12.37	13.52	13.99	13.95
	R^2	0.959	0.982	0.985	0.970	0.986	0.964	0.977	0.979	0.944	0.979	0.998	0.998
	χ^2	0.004	0.004	0.001	0.095	0.107	0.105	0.0693	0.156	0.737	0.135	0.006	0.009

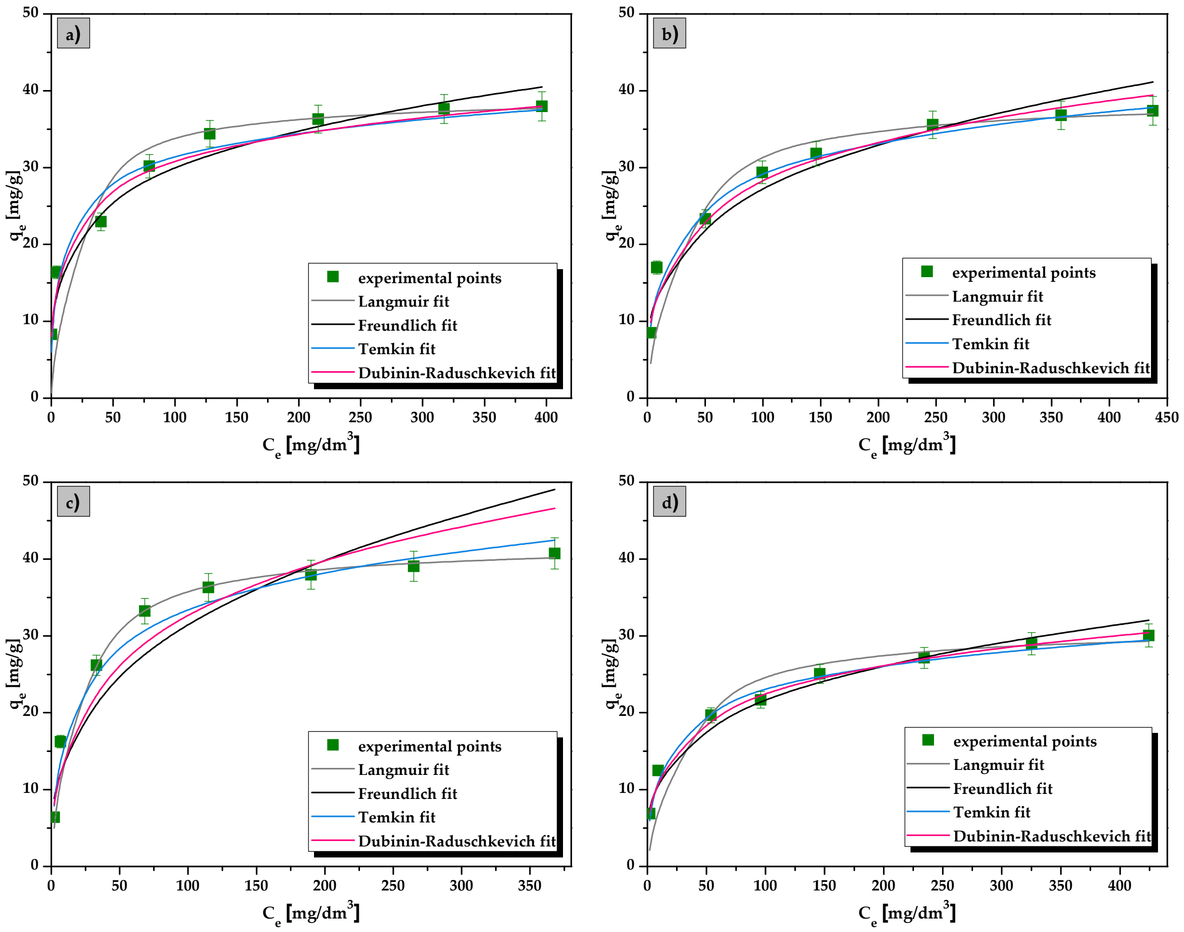

Figure S8. Nonlinear fitting of isotherm models for (**a**) La(III), (**b**) Ce(III), (**c**) Pr(III), and (**d**) Nd(III) ions sorption on raw lignin at 333 K.
